# Effects of silicon carbide nanoparticles on mechanical and vibrational characteristics of carbon glass epoxy hybrid composites

**DOI:** 10.1038/s41598-026-39559-4

**Published:** 2026-02-09

**Authors:** K. S. Suhas, Vamsi Krishna Reddy, Yeturi Thirumanas Reddy, Yogeesha Pai

**Affiliations:** Manipal Institute of Technology, Manipal Academy of Higher Education (MAHE), Manipal, India

**Keywords:** SiC nanoparticle, Glass-carbon hybrid laminate, Mechanical testing, Damping ratio, Acoustic properties, Natural frequency, Engineering, Materials science, Nanoscience and technology

## Abstract

This study presents an experimental investigation into the mechanical, vibrational, and acoustic properties of carbon/glass fibre-reinforced epoxy hybrid laminates embedded with varying weight percentages of silicon carbide (SiC) nanoparticles. The laminates were fabricated using compression moulding, with a six-layer alternating stacking sequence of carbon and glass fibres. A comprehensive series of tests was conducted to assess flexural strength, tensile behaviour, impact resistance, free-vibration characteristics, and sound-absorption performance. Mechanical tests revealed that the laminate with 3 wt% SiC achieved the highest tensile strength of 258.8 MPa, flexural strength of 292.6 MPa, Young’s modulus of 19.13 GPa, and impact strength of 67.9 kJ/m^2^, indicating optimal reinforcement and efficient stress transfer due to uniform nanoparticle dispersion. These values correspond to improvements of approximately 19.05%, 15.22%, 15.37%, and 7.65%, respectively, compared to the unreinforced (0 wt%) composite. SEM analysis substantiated the improved fibre–matrix interaction and the minimal microstructural defects at the optimal filler content. Conversely, the 5 wt% SiC specimens exhibited reduced mechanical performance, attributed to particle agglomeration and weakened interfacial bonding. Vibration analysis indicated a peak in stiffness and natural frequency at 3 wt% SiC, while damping behaviour declined with increasing filler loading. Acoustic testing showed enhanced transmission loss with increasing SiC content, with 5 wt% yielding the best sound-attenuation performance. The study concludes that incorporating SiC nanoparticles into carbon/glass hybrid composites significantly improves their multifunctional performance when the filler content is optimized, with the 3 wt% SiC composition offering the best balance between strength, stiffness, and acoustic efficiency.This advances SDG 9 (Industry, Innovation and Infrastructure) by developing resilient, lightweight composites for sustainable aerospace/transport infrastructure, reducing emissions via efficiency gains.

## Introduction

Composites are advanced materials engineered by combining two or more constituent materials with distinct physical and chemical properties to achieve superior mechanical performance. Their excellent properties make them essential in various industries, including aerospace, automotive, marine, and construction^1,2^. Polymer matrix composites (PMCs) offer high stiffness, resistance to corrosion, and low weight, providing significant advantages over conventional materials. Hybrid composites represent a further advancement in composite technology, incorporating two or more types of reinforcing fibres within a single matrix to achieve a balanced combination of mechanical, thermal, and durability properties^3^.

One of the most widely used synthetic fibres in the aviation industry is carbon fibre owing to its advantages such as high tensile strength and lightweight properties^4^. Due to their high price tags and longer moulding cycle times, these materials are generally reinforced with glass or other fibres, better suited for lower-volume manufacture. Glass fibres are synthetic fibres which are well known for their high strength, excellent durability, corrosion resistance, and lightweight properties^5^. Hybridising carbon with glass fibres within a polymer matrix is used to achieve a balance between cost, strength, and weight. Several studies highlight the positive effects of hybridising glass and carbon fibres, demonstrating improved overall performance in composite materials^6,7^.

Nanotechnology has been a well-established field of study since the previous century. The concept of nanotechnology was first put forth by Nobel Prize winner Richard P. Feynman in his famous 1959 address, *There’s Plenty of Room at the Bottom*^8^. Nanoparticles are a broad class of materials with at least one dimension smaller than 100 nanometers^9^. Research has also examined how various nanoparticle fillers affect the mechanical and tribological properties of epoxy-based nanocomposites^10–12^. These studies often highlight the importance of filler loading levels, with nanofillers typically enhancing composite performance more consistently than micron-scale fillers. Increasing filler content improves mechanical and wear properties up to a critical threshold, beyond which performance declines primarily due to the agglomeration of nanofillers in the resin matrix^13–15^. Silicon carbide (SiC) nanoparticles are composed primarily of silicon (Si) and carbon (C) atoms arranged in a crystalline structure. The typical composition includes silicon (50%) and carbon (50%). These nanoparticles exhibit high hardness, excellent wear resistance, and superior thermal stability, making them ideal for applications in aerospace, automotive, electronics, and composite materials.

Table 1 delivers the outline of the various existing literature that examines the effect of nanofillers on composite materials. Numerous studies have explored the influence of nanofillers on the mechanical and thermal performance of fibre-reinforced polymer composites. Carbon nanotubes (CNTs) were incorporated into a carbon fibre–epoxy resin system, resulting in a significant enhancement of both flexural modulus and strength, attributed to the superior reinforcement potential of CNTs^16^. Similarly, silica nanoparticles, when added to a glass fibre and dental fibre-reinforced composite resin, significantly altered the mechanical behaviour and improved fibre impregnation during the resin phase^17^. In terms of thermal performance, increasing the silicon carbide (SiC) filler content in glass fibre–epoxy resin composites improved thermal conductivity, particularly beyond a 15% filler fraction^18^. Graphene oxide (GO) has been extensively studied for its reinforcing effects on bio-epoxy composites and was found that a 0.3 wt% GO filler yielded the most favourable mechanical properties. However, higher concentrations reduced performance, likely due to agglomeration effects^19^. In another study, enhancement was observed in the flexural and tensile strength of Glass/Kevlar-epoxy composites, with optimum properties achieved at 5 wt% of graphene oxide^20^. Rajesh et al.^21^ explored the effect of tungsten carbide as a filler in aluminum alloy 6061 matrix composites, and concluded that a 3% filler content was ideal for improving mechanical characteristics. Girimurugan et al.^22^ experimented with the influence of nano-alumina in carbon fibre-epoxy resin systems, where an increasing filler percentage from 1% to 5% improved the mechanical behaviour. However, filler concentrations beyond 3–4% began to negatively affect matrix properties due to possible poor dispersion and increased brittleness. Huang et al. investigated glass fibre–polyester resin composites with aluminum powder fillers and found that adding 2% aluminum powder reduced hardness, whereas increasing the filler content to 5% and 10% led to significant improvements in the composite’s hardness^23^.

**Table 1:** Literature study on effect of nanofillers on composite.

Published Year and Reference	Type of composite	Nanofiller used	Weight% (wt%)	Findings
2004^24^	Carbon/ epoxy	Silicon carbide	1.5 to 3	1.5 wt% of filler resulted in an enhancement of 20–30% in mechanical characteristics.
2013^16^	Carbon fibre, epoxy resin	Carbon Nanotubes	0.1, 0.2, 0.3	Composites with a CNT-modified matrix have higher flexural modulus and strength values.
2016^17^	Glass fibre, Dental fibre reinforced composite resin	Silica	0, 0.2, 0.5, 2,5	SiO_2_ nanoparticles significantly changed the mechanical behaviour of the FRP composite and improved fibre impregnation when added to the resin phase.
2019^25^	E-glass/epoxy with SiO₂ and carbon NPs	SiO₂ (with/without Carbon)	0.25 to 1	Tensile strength and fatigue life improved with nano-filler addition. A 19% increase in tensile strength and a 60% in fatigue life were observed at 0.5 wt% carbon. The Simultaneous addition of SiO₂ and carbon improved modulus and impact strength.
2019^26^	Glass Fiber, Epoxy resin	Silicon Carbide	5, 10, 15, 20, 25	Thermal conductivity was shown to rise as the SiC filler fraction increased, and it rose noticeably above 15% SiC filler fraction.
2021^19^	Bio epoxy	Graphene Oxide	0.1, 0.2, 0.3, 0.6	The ideal amount of GO filler was found to be 0.3 wt%, since this increased the tensile strength, tensile modulus, flexural strength, and flexural modulus.
2020^21^	Aluminium alloy 6061 matrix	Tungsten Carbide	1, 2, 3, 4	Three wt% of the reinforcing mix was found to increase the mechanical characteristics.
2023^22^	Carbon fibre, epoxy resin	Nano Alumina	1, 2, 3, 4, 5	The mechanical characteristics of the produced composite specimens improved as the filler particle wt% increases. The mechanical properties of the epoxy matrix decreased with an increase in the amount of filler (3, 4, and 5 wt%) substances.
2023^27^	Carbon/Basalt, Polyester resin	Silicon Carbide	0, 5, 10, 15	The tensile, flexural, and impact resistance of a polyester matrix containing 15 wt% SiC nanoparticles were significantly higher than those of matrices containing 0, 5, and 10 wt%.
2023^28^	Carbon fiber/epoxy with Al₂O₃ and SiC NPs	Silicon carbide	1, 1.25, 1.5, 1.75 and 2	Superior tensile and hardness properties were observed for carbon fibre composites with Al₂O₃ at 1.75 wt% and SiC nanofillers at 1.25 wt% loading compared to neat composites.

Recent advancements in polymer nanocomposites have shown that nanofillers such as graphene, Al₂O₃, SiO₂, and CNTs significantly influence the multifunctional behaviour of epoxy systems through improved interfacial bonding and dispersion control. A study has demonstrated that statistically optimized graphene/epoxy nanocomposites achieved remarkable mechanical and thermal enhancements even at ultralow filler loadings, emphasizing the importance of interfacial engineering and processing optimization^29^. Similar approaches have been reported for other nanofillers, where controlled dispersion and surface modification play a decisive role in achieving optimal reinforcement efficiency. These findings reinforce the relevance of the present study, which explores SiC nanoparticle incorporation under comparable reinforcement principles. The collective literature indicates that both the type and percentage of nanofillers significantly influence the mechanical, thermal, and physical characteristics of FRP composites. Optimal filler concentrations vary by nanofiller type, with most studies identifying a threshold beyond which mechanical performance declines. Graphene oxide, silicon carbide, and nano-alumina are particularly effective at improving strength and modulus within their optimal loading ranges. These findings highlight the importance of precise filler selection and loading to tailor composite properties for specific applications.

Despite extensive research on nanofillers in polymer composites, there remains a gap in understanding the effects of silicon carbide (SiC) nanofillers on carbon/glass hybrid composites, particularly regarding their mechanical and vibrational properties. The synergistic effects of combining SiC nanoparticles with alternating carbon and glass fibre layers in a hybrid composite matrix have not been fully explored. The developed carbon–glass epoxy hybrid composite reinforced with silicon carbide (SiC) nanoparticles demonstrates significant potential for use in aerospace and automotive structures where high mechanical strength, vibration damping, and impact resistance are essential. Improved sound absorption and vibrational performance also make these materials suitable for noise-reduction panels, engine covers, and interior structural components that require both durability and acoustic insulation.

This study addresses the identified research gap by investigating the effect of silicon carbide (SiC) nanoparticles (0–5 wt%) on carbon-glass fibre hybrids arranged in an alternating six-layer stacking sequence, with epoxy resin as the matrix. The objectives include fabricating carbon–glass epoxy hybrid composites with 1 wt%, 3 wt%, and 5 wt% SiC particles; conducting mechanical and vibrational tests (tension, flexural, impact, free vibration, and sound absorption); and analyzing damage morphology using Scanning Electron Microscopy (SEM). Hybrid laminates are fabricated using compression moulding, and all tests are conducted according to ASTM and ISO standards. Unlike existing studies that primarily focus on either mechanical or thermal performance of nanofiller-reinforced composites, this work presents a comprehensive multifunctional evaluation of silicon carbide (SiC) nanoparticle-reinforced carbon–glass hybrid epoxy laminates, integrating mechanical, vibrational, and acoustic behavior within a single experimental framework. The novelty of this study lies in (i) the systematic correlation between SiC nanoparticle loading and simultaneous changes in stiffness, damping, and sound attenuation, (ii) the identification of an optimal filler concentration (3 wt%) based on combined structural and dynamic performance rather than strength alone, and (iii) the use of SEM-supported failure mechanism analysis to explain the transition from reinforcement-dominated behavior to agglomeration-induced degradation at higher filler contents. These insights provide mechanistic understanding relevant to lightweight structural components requiring both mechanical integrity and vibration/noise control.

## Materials and fabrication

### Materials

**Fig. 1:**
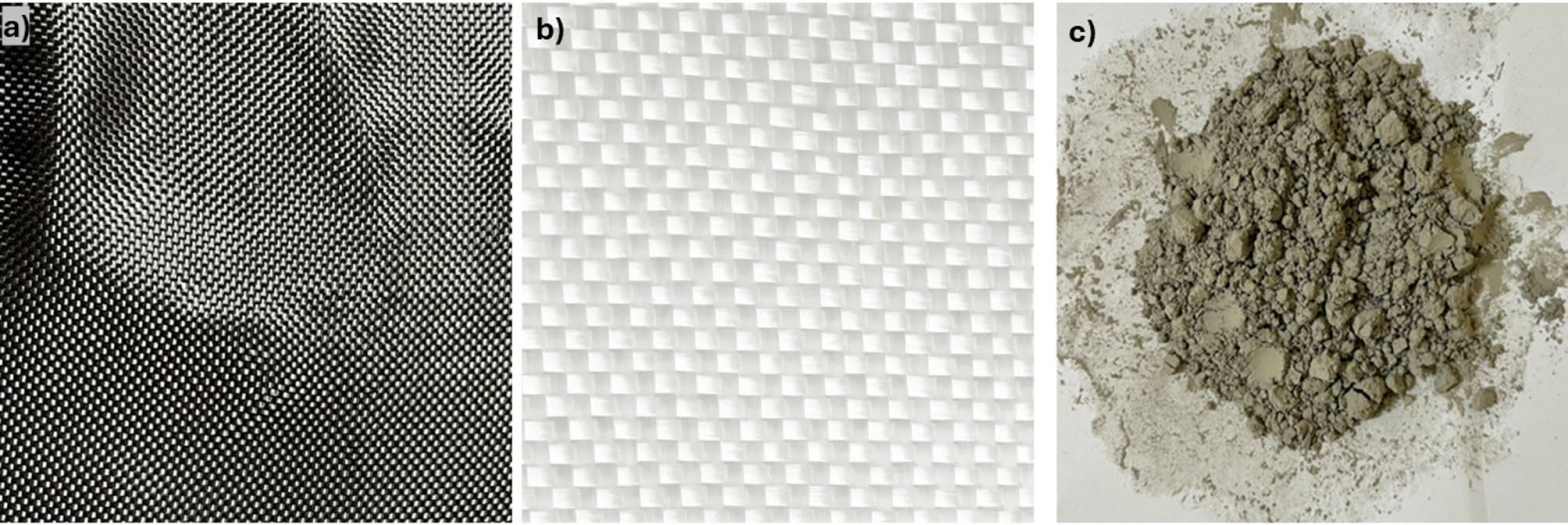
Images of (**a**) carbon fibre (**b**) glass fibre (**c**) SiC nanoparticles.

Bi-directional glass and carbon fibres of 300 GSM and 200 GSM, respectively, were procured from Bhor Chemicals and Plastics Pvt. Ltd., Maharashtra, India. Epoxy resin (BhorBond^®^ EPCH) and hardener (BhorBond^®^ EPCH) were used as the composite matrix. SiC nanoparticles were used as the composite fillers. The SiC nanoparticles utilized have a chemical composition of silicon carbide (SiC), comprising silicon and carbon elements. The particle size distribution ranges from approximately 10 to 100 nm, with an average particle size around 42 nm, as characterized by TEM and XRD analyses. Structurally, the nanoparticles are polycrystalline with crystalline domains averaging about 21 nm, exhibiting typical features such as twins and stacking faults. These properties ensure their suitability for reinforcing composite materials and contribute to the mechanical and thermal characteristics observed in the study. The image of the fabric and SiC nanoparticles is shown in Fig. 1. The properties of glass fibre, carbon fibre, and matrix material are shown in Tables 2 and 3, respectively.

**Table 2:** Reinforcement properties.

Reinforcement	Elastic modulus (GPa)	Density (g/cc)	Weave type
Carbon	200	1.9	Bi-directional
Glass	72	2.5	Bi-directional
SiC	70.25	1.7	Particulate

**Table 3:** Matrix properties.

Matrix components	Brand	Viscosity (cP)	Density (g/cc)
Epoxy resin	BhorBond^®^ EPCH	11,500–13,500	1.15–1.2
Hardner	BhorBond^®^ EPCH	˃100	0.94–0.95

### Fabrication

**Fig. 2:**
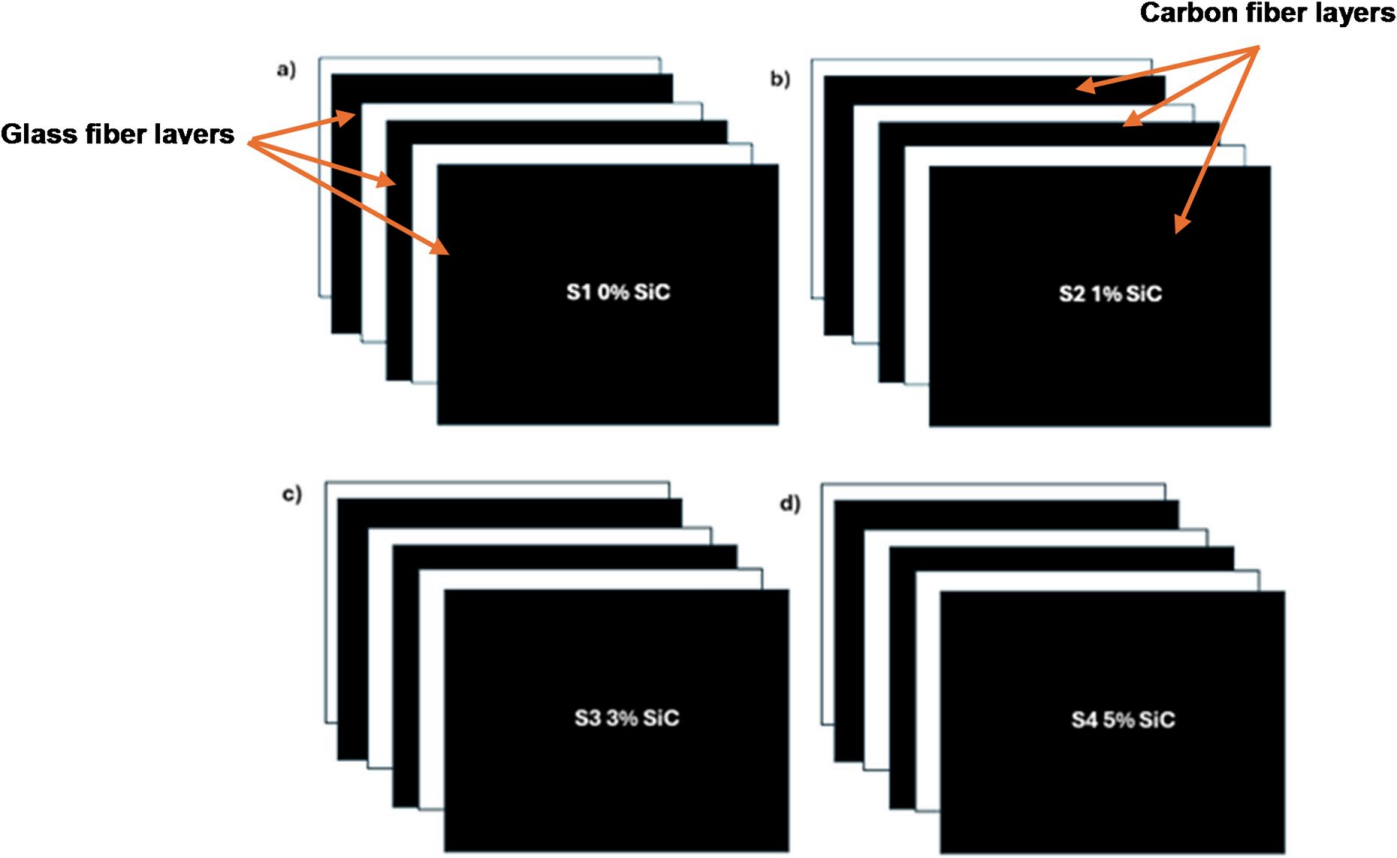
Stacking sequence of laminates with different wt% of SiC.

SiC nanoparticles as a filler and woven bidirectional mat as reinforcement made of carbon and glass fibers were used to create laminates (300 mm × 300 mm × 2 mm). Six layers of bidirectional reinforcement were used in the construction of the laminate. Figure 2 shows the stacking sequence and % of SiC nanoparticles (0, 1, 3 and 5%) employed in the manufacturing process. Black-coloured layers represent carbon fiber, and the white-coloured layers represent glass fiber, arranged in an alternating sequence to illustrate the hybrid laminate layup.

**Fig. 3:**
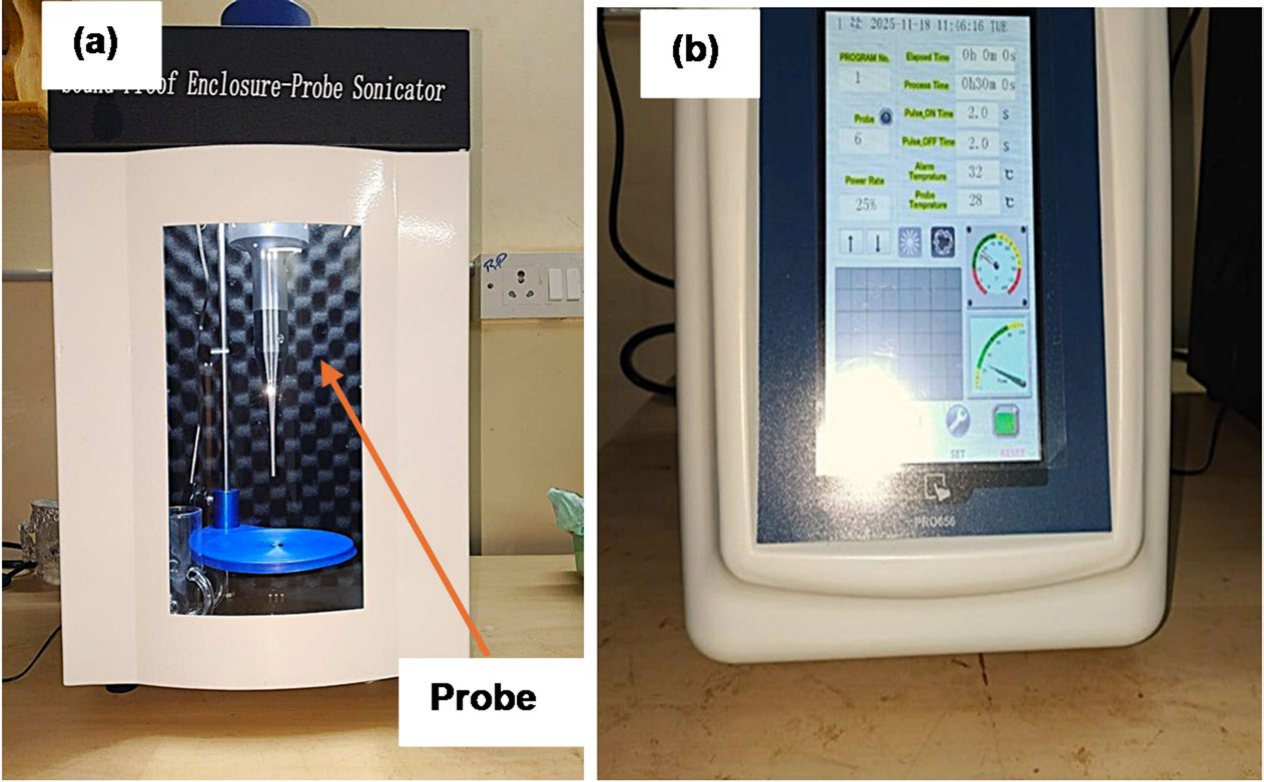
(**a**) Probe Sonicator for SiC Dispersion (**b**) Control panel.

A 300 mm × 300 mm open mild steel plate served as the base for laminate fabrication using the conventional hand lay-up technique. To ensure a smooth and defect-free surface, all plates were thoroughly cleaned and coated with a release agent before the layup, facilitating easy removal of the cured laminate. On the other hand, the SiC nanoparticles were dispersed in epoxy resin by mechanical stirring for 120 min to achieve uniform distribution. The mixture was then subjected to sonication for 30 min to further enhance nanoparticle dispersion. A probe sonicator was used to ensure effective dispersion of SiC nanoparticles in the epoxy matrix by delivering high-frequency ultrasonic energy directly into the mixture, breaking particle clusters and promoting uniform distribution. The device operates in the frequency range of 20–25 kHz with an adjustable power capacity from 6.5 to 650 W, and supports processing volumes between 0.2 and 500 mL. It is equipped with a 6 mm standard probe, a 7-inch touchscreen display, and features real-time temperature and power monitoring with an operating temperature range from 0 to 1000 °C. The system includes programmable ultrasound timing (0.1 to 99.9 s) and multiple built-in safety alarms such as overload, no-load, and over-temperature alerts. These specifications ensured controlled and consistent sonication during nanoparticle dispersion.Sonication instrument setup is shown in Fig. 3.

**Fig. 4:**
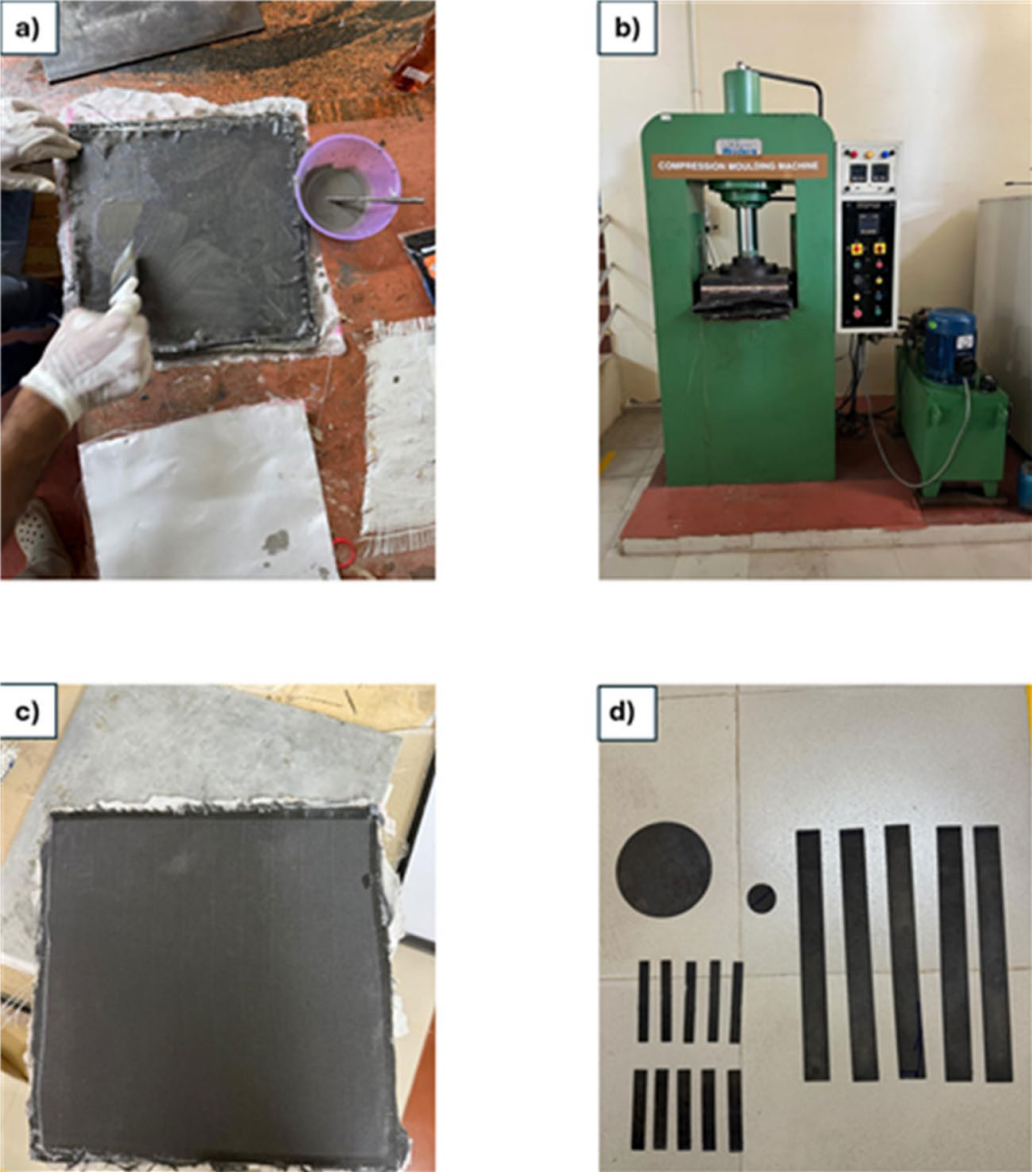
Details of fabrication process (**a**) hand lay-up, (**b**) compression molding, (**c**) cured laminate (**d**) cut specimens.

After sonication, the mixture was placed in a vacuum chamber for 30 min to remove entrapped air bubbles. Following degassing, the appropriate amount of hardener (100:35) was added and thoroughly mixed with the nanoparticle-epoxy mixture using a spatula to ensure complete homogenization prior to composite fabrication. The fiber-to-resin ratio was maintained at 60:40 by weight for all samples. The prepared SiC nanoparticle-reinforced epoxy mixture was applied to the fabrication of carbon-glass fiber composites using the hand lay-up technique. After the layup, the laminate was transferred to a compression molding machine equipped with spacers to achieve a laminate of 2 mm thickness. The curing process was carried out at a temperature of 120 °C under a uniform pressure of 50 bar. The laminate was held under these conditions for 60 min to ensure complete resin flow, fibre wetting, and nanoparticle dispersion within the matrix. After curing, the mould was allowed to cool gradually to room temperature under pressure to prevent warpage and residual stresses. The composite was permitted to cure at ambient conditions for 1 day. After curing, the laminates were marked and cut according to ASTM and ISO standards using abrasive water jet machining (AWJM) techniques. The detailed fabrication steps are illustrated in Fig. 4.

## Experimental method

All mechanical and vibrational tests were conducted in accordance with the respective ASTM and ISO standards, as referenced in the following subsections, to ensure reproducibility and reliability of the obtained results.

### Void fraction

Void content refers to the presence of air pockets or unfilled regions within a laminate, which can significantly weaken mechanical properties. High void content reduces strength and fatigue resistance, and is often caused by improper curing, resin flow issues, or poor fiber wetting. The density of the laminates was determined as per ASTM D792-20^30^. The square specimens of side 10 mm were cut, and the density was measured using Archimedes’ principle. The mass of each specimen was calculated using an electronic weighing scale. The sample’s volume was obtained using the volume of water displaced. The mass-to-volume ratio, which is the experimental density, was then calculated. Five samples were taken from different parts of the composite, and the mean density was computed. Theoretical densities were calculated by applying Eq. (1).

1 $$\:{\rho\:}_{th}=\frac{1}{\frac{{w}_{f}}{{\rho\:}_{f}}+\frac{{w}_{m}}{{\rho\:}_{m}}}$$

Equation (2) was used to calculate the laminate’s void percentage.

2 $$\:Void\left(\mathrm{\%}\right)=\frac{{\rho\:}_{th}-{\rho\:}_{ex}}{{\rho\:}_{ex}}\times\:100$$

Here, $\:{w}_{f}$ signifies the fibre weight fraction and $\:{w}_{m}$ signifies matrix weight fraction. $\:{\rho\:}_{th}$ signifies the theoretical density and $\:{\rho\:}_{ex}$ experimental density.

### Tensile test set up

**Fig. 5:**
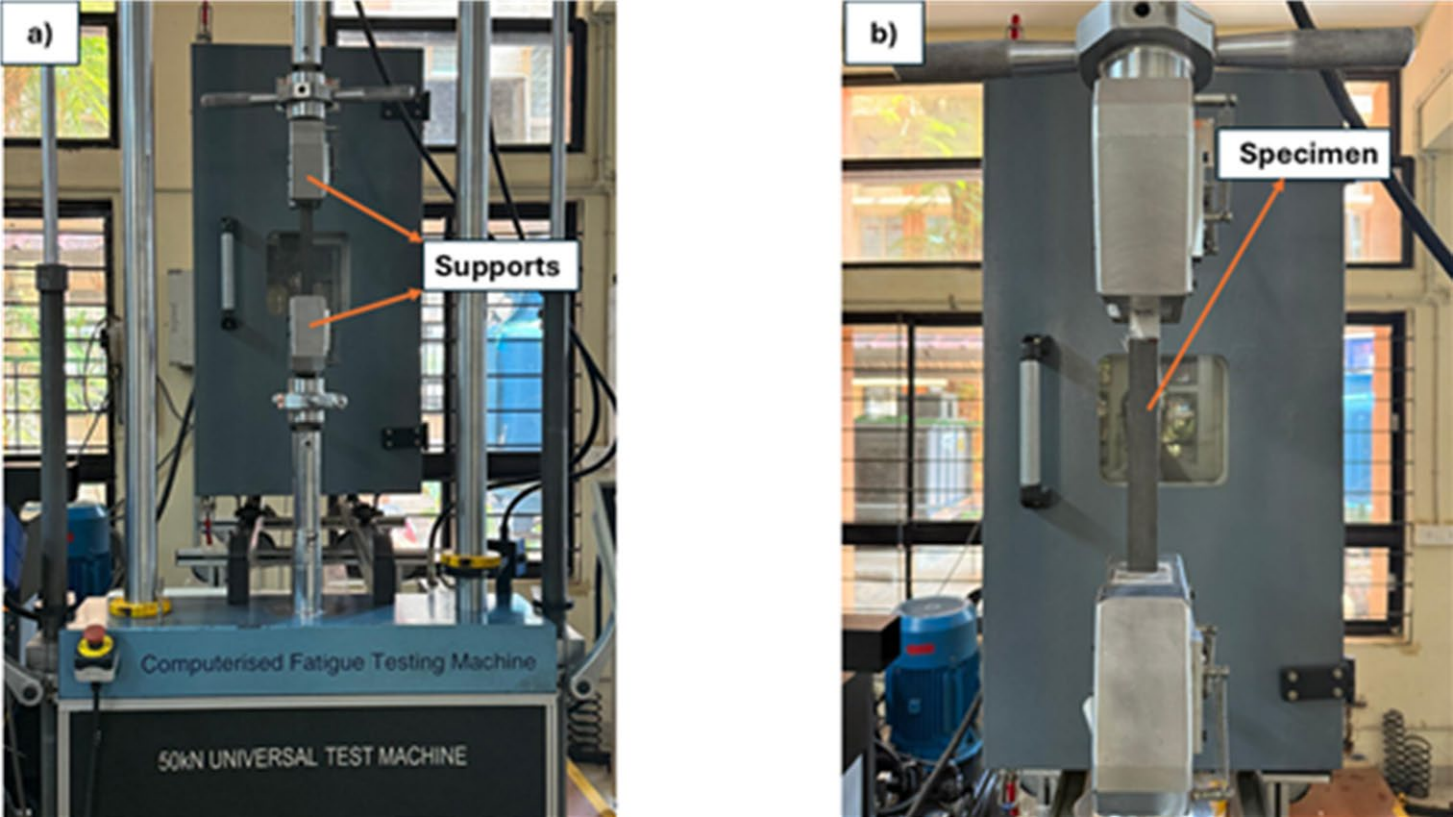
Tensile test setup.

A tensile test is a mechanical test where a material is stretched until failure to determine its strength, ductility, and elasticity, essential for designing components in aerospace, automotive, construction, and biomedical industries. This test provides key data such as ultimate strength, elastic modulus, and elastic strain at rupture. Five specimens were tested for each composition (0, 1, 3, and 5 wt% SiC) in accordance with ASTM D3039^31^. Testing was performed on a 50 kN loading capacity Universal Testing Machine (BiSS), as shown in Fig. 5. Each specimen was loaded at a constant crosshead speed of 2 mm/min, with a fixed gauge length of 190 mm between the grips, until failure occurred.

### Flexural test

**Fig. 6:**
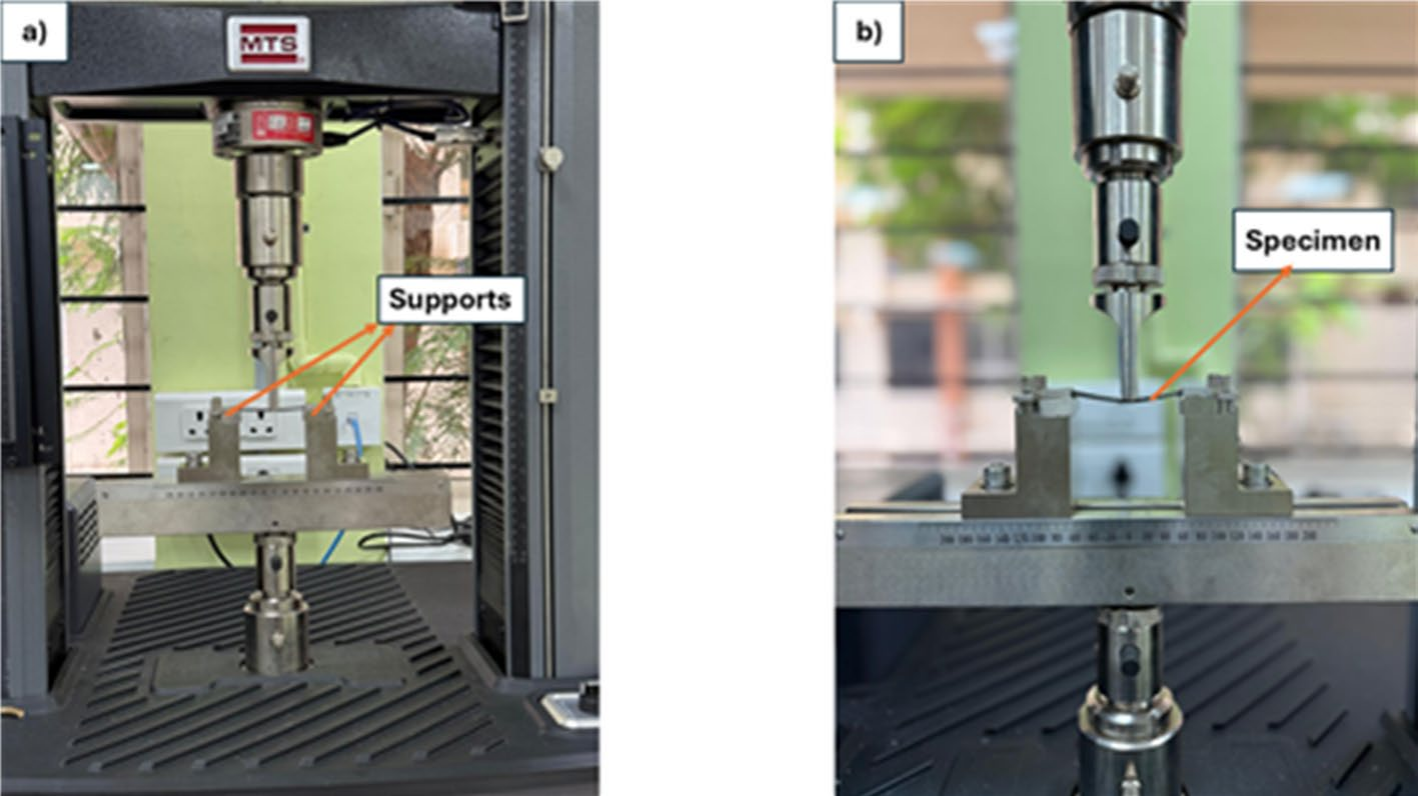
Flexural test set up.

The flexural test was conducted to assess the bending resistance of the specimen before permanent deformation, following the ASTM D7264-48^32^ standard. Specimens with dimensions of 76.4 mm × 13 mm were prepared, maintaining the standard ratio of span to thickness of 32:1. The specimen was simply supported on both span edges and loaded at the midspan as per three-point bending method. Five specimens per composition were tested to ensure statistical reliability. As seen in Fig. 6, the test was conducted with the help of an MTS Electromechanical UTM, Exceed Model E43 of 50 kN maximum loading capacity. A constant crosshead displacement rate of 2 mm/min was used during the test until failure, with the gauge length between the supports fixed at 64 mm. Following testing, the loading and displacement were calculated and then transformed into flexural stress ($\:{\sigma\:}_{f}$) and flexural strain ($\epsilon$) using Eqs. (3) and (4) ,respectively.

3 $$\:{\sigma\:}_{f}=\frac{3FL}{2b{d}^{2}}$$

4 $$\epsilon =\frac{6dD}{{L}^{2}}$$

### Charpy impact test

**Fig. 7:**
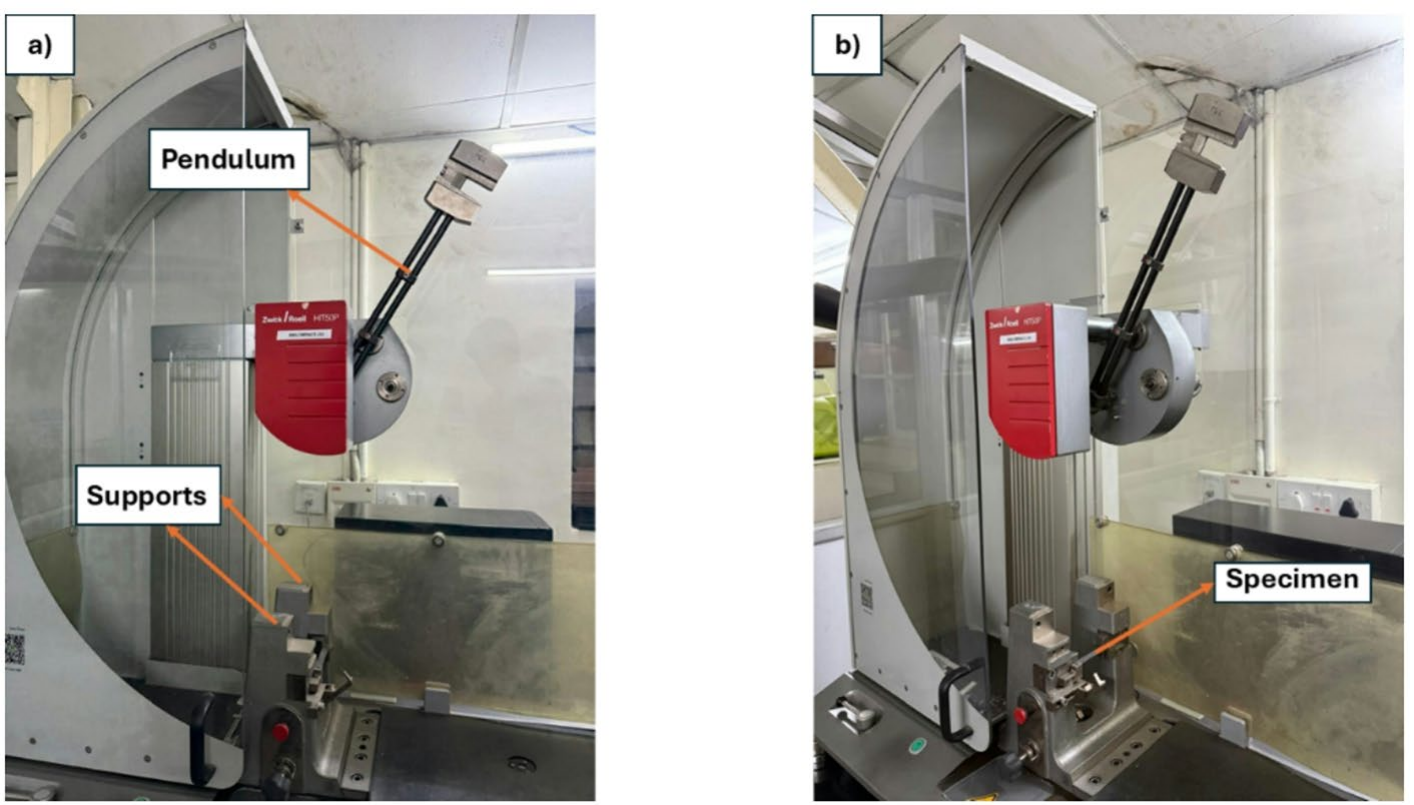
Impact test setup.

A widely used technique for assessing the toughness of a material and resistance to sudden forcible impacts is the Charpy impact test. Specimens measuring 80 mm × 10 mm were examined as per ISO 179–1150^33^ with five samples from each category. For analysis, the mean results of these tests were taken into account. A Zwick/Roell Hit 50P machine, which has a working capacity of 25 J and a theoretical impact velocity of 3.807 m/s, was used for the experiments. As shown in Fig. 7, the testing apparatus consists of a high-stiffness pendulum, elevated to a predefined height and then released to strike a certain place on the specimen. The specimens were securely positioned to ensure the center of impact was located along a 32 mm span length.

### Free vibration test

**Fig. 8:**
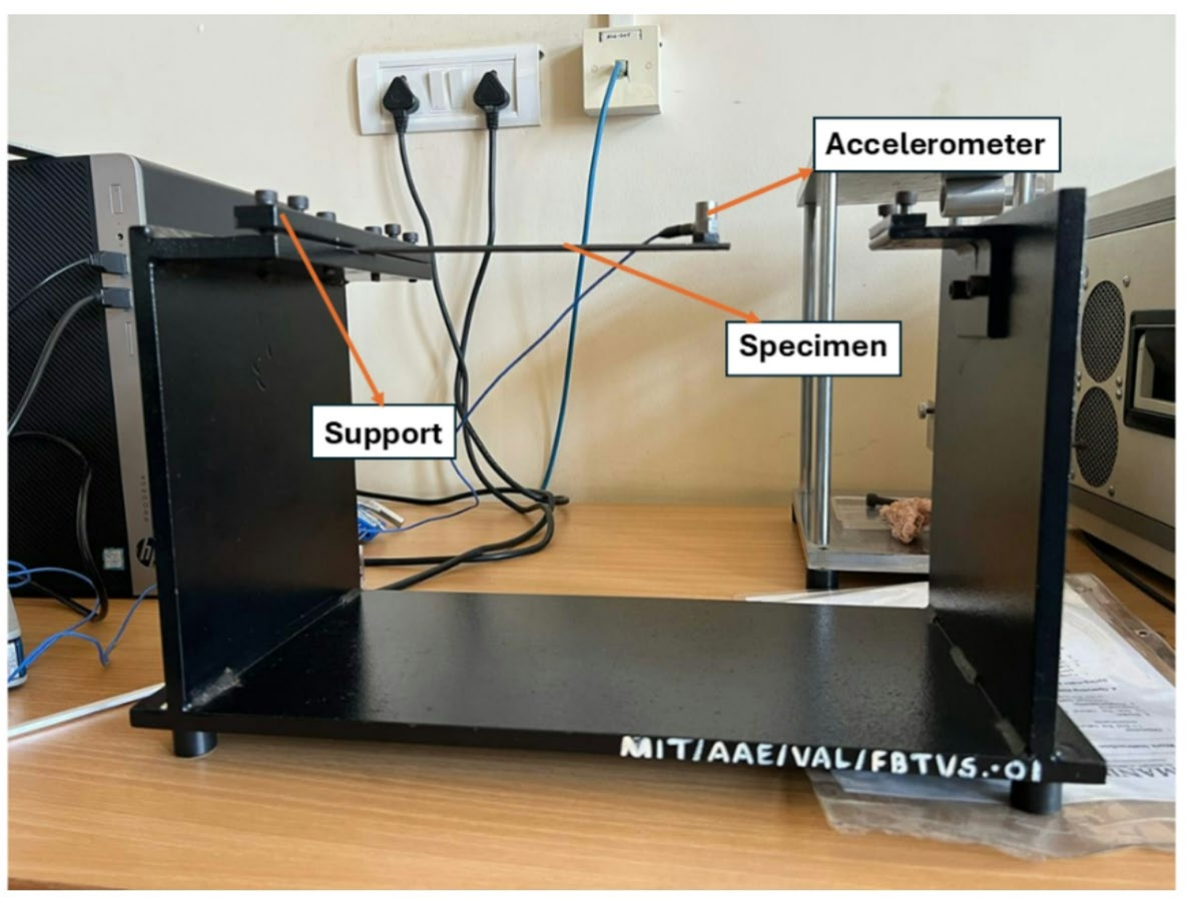
Free vibration test set up.

Vibrational analysis plays a vital role in understanding the natural dynamic behaviour of a material when disturbed without continuous external force. It helps in understanding significant properties like stiffness, natural frequency, and the amount of energy lost through damping. To study these properties, tests were carried out following the ASTM E756-05^34^ standard. The test samples were made to specific dimensions of 250 mm long and 25 mm wide. As shown in Fig. 8, each sample was set up like a cantilever beam, with one end fixed and the other free to move. This is because of repeatability, simplicity, and reduced boundary uncertainty. A highly sensitive PCB Piezotronics accelerometer (106.3 mV/g) was attached to the free end to capture vibration data. The specimen in the impact hammer test was excited using a calibrated modal hammer with a sensitivity of 10.2 mV/lbf to apply controlled impulse forces. The dynamic response was captured using the National Instruments NI-9234, a 4-channel, 24-bit high-resolution DAQ module designed for IEPE sensors, providing simultaneous sampling up to 51.2 kS/s per channel and built-in IEPE excitation to power the accelerometers. From the recorded data, the stiffness (K), damping ratio (ζ), and storage modulus (E_s_) were calculated using standard formulas provided in Eqs. (5), (6), and (7).

5 $$\:k=\frac{3{E}_{s}I}{{L}^{3}}$$

6 $$\:{f}_{n}=\frac{1}{2\pi\:}\sqrt{\frac{k}{m}}$$

7 $$\:{E}_{s}=\frac{16{\pi\:}^{2}{f}_{n}^{2}m{L}^{3}}{b{h}^{3}}$$

The suitability of a material for a structural application can be determined in this test by analyzing the dynamic behavior of the material using the above equations. The logarithmic decrement and the damping ratio are two important parameters which assist in understanding how fast oscillations die out over time. The logarithmic decrement, denoted as δ, provides a measure of how quickly the system loses energy, and it is analyzed by comparing the amplitudes of two successive peaks in the vibration, with X₁ as the first peak and X₂ as the second, as shown in Eq. (8).

8 $$\:\delta\:=\frac{1}{n}\mathrm{l}\mathrm{n}\left(\frac{{X}_{1}}{{X}_{2}}\right)$$

The damping ratio (ζ) is a dimensionless number that describes how quickly the vibrations decay after a disturbance. It is measured in terms of the logarithmic decrement, as shown in Eq. (9).

9 $$\:\zeta\:=\frac{\delta\:}{\sqrt{4{\pi\:}^{2}+{\delta\:}^{2}}}$$

### Sound absorption test

**Fig. 9:**
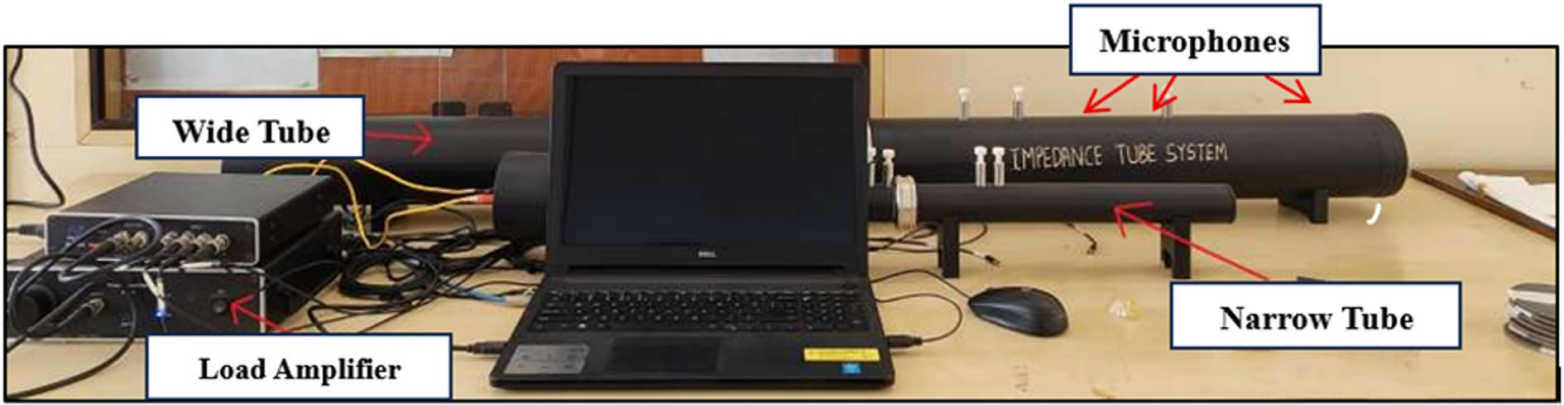
Impedance tube test setup.

Transmission loss refers to the reduction in acoustic energy as sound passes through a material. It’s measured in decibels (dB) and represents the difference in sound pressure levels between the incoming sound and the sound that makes it through. This property is especially important in acoustics, as it helps evaluate and design materials that can effectively block or reduce unwanted noise. To measure transmission loss in this study, the impedance tube method was used, following the procedures outlined by BSWA Technology and the ISO 10534-2^35^ standard. As shown in Fig. 9, circular specimens with diameters of 99.5 mm and 29.5 mm were tested. To capture the sound behavior, four microphones with different sensitivities were placed at specific positions in both the large and small tubes. The test ran for about ten minutes, covering a frequency range from 63 Hz up to 6300 Hz, using the transfer function method **t**o analyze the data. By combining the results from both tubes, a graph was created showing transmission loss versus frequency. This gave a clear picture of how well the material blocks sound across different frequencies. The insights gained from this method are essential for designing materials with better sound insulation and noise reduction capabilities useful in everything from construction to automotive and industrial applications.

## Results and discussion

### Void content

Table 4 shows the void percentages for composites with different SiC wt%. All values were within the acceptable industrial limit of 5%, indicating good quality of fabrication. The void content decreased steadily with increasing SiC content—from 2.61% at 0 wt% to just 1.06% at 5 wt%. This reduction suggests that the addition of SiC particles helped improve matrix packing and reduce porosity. However, while lower voids generally enhance mechanical properties, the 5 wt% sample showed reduced strength, indicating that factors like particle agglomeration and poor interfacial bonding may offset the benefits of reduced voids beyond an optimal filler content.

**Table 4:** Void content results of composites.

SiC wt%	Void Percentage
0	2.61%
1	2.05%
3	1.64%
5	1.06%

### Tensile behaviour

**Fig. 10:**
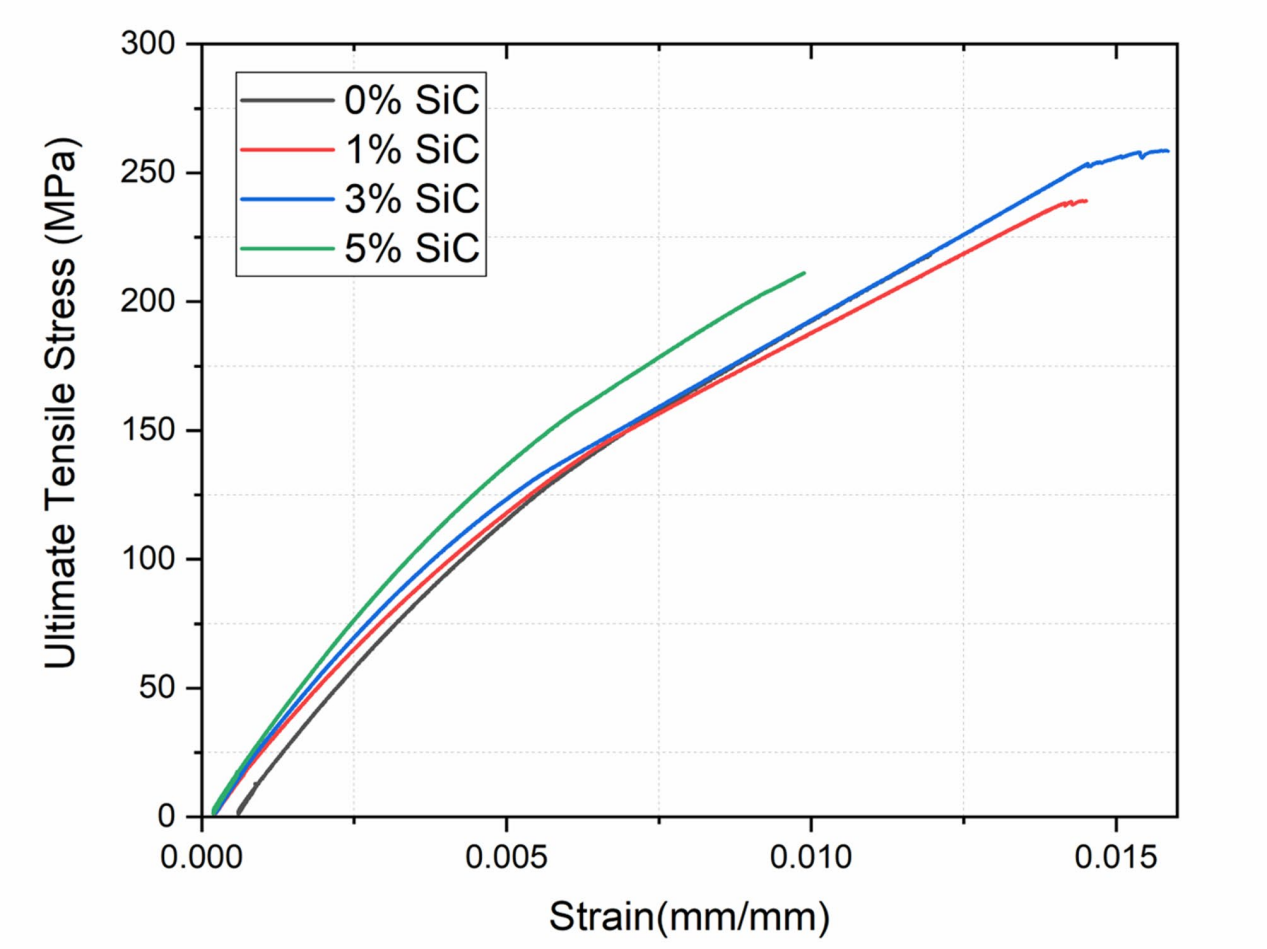
Representative stress–strain curves of composites at different SiC loadings, showing the increase in stiffness and ultimate stress up to 3 wt% SiC.

Table 5 summarizes the tensile behavior of the composite specimens with varying wt% of silicon carbide (SiC) reinforcement, specifically 0%, 1%, 3%, and 5%. The results provide insight into how SiC content influences the tensile strength, stiffness, and ductility of the composite material. Figure 10 shows the corresponding stress-strain plots of the composites.

**Table 5:** Tensile properties of composites with varying SiC content.

SiC wt%	Max Tensile Load (kN)	Ultimate Tensile Strength (MPa)	Ultimate Tensile Strain	Young’s Modulus (GPa)
0	10.86	217.37 ± 6.52	0.0119	16.58 ± 0.70
1	11.96	239.72 ± 7.19	0.0140	17.91 ± 0.75
3	12.94	258.80 ± 7.76	0.0158	19.13 ± 0.81
5	10.53	210.70 ± 6.32	0.0098	22.09 ± 0.89

The specimen without nanofiller (0 wt% SiC) served as the baseline, exhibiting a maximum tensile load of 10.86 kN, an ultimate tensile strength of 217.37 MPa, and a tensile strain of 0.0119. The corresponding Young’s modulus was found to be 16.58 GPa. With the incorporation of 1 wt% SiC, an increase in performance was observed. The tensile strength increased to 239.72 MPa and Young’s modulus to 17.91 GPa, which is an increment of 10.28% and 8.02%, respectively, compared to 0 wt% SiC composites. Further increasing the filler to 3 wt% SiC, a notable enhancement in tensile behavior was recorded. The composite achieved its highest ultimate tensile strength of 258.8 MPa. Comparable to the baseline material, a strain of 0.0158 was achieved, indicating that the ductility was maintained while strength improved. The Young’s modulus at this stage was 19.13 GPa, suggesting that the stiffness of the material also benefited from the uniform distribution and effective load transfer from matrix to reinforcement at this optimal SiC concentration. However, increasing the SiC content further to 5 wt% showed a decline in tensile performance. The ultimate tensile strength decreased drastically to 210.7 MPa, and the maximum tensile load dropped to 10.53 kN. The tensile strain also showed a sharp reduction to 0.0098, indicating a substantial loss in ductility. Remarkably, Young’s modulus continued to increase, reaching 22.09 GPa, the highest among all compositions. This trend suggests that although stiffness improved, the material became increasingly brittle and less capable of sustaining tensile loads, likely due to agglomeration of SiC particles or microstructural defects that acted as crack initiation sites.

**Fig. 11:**
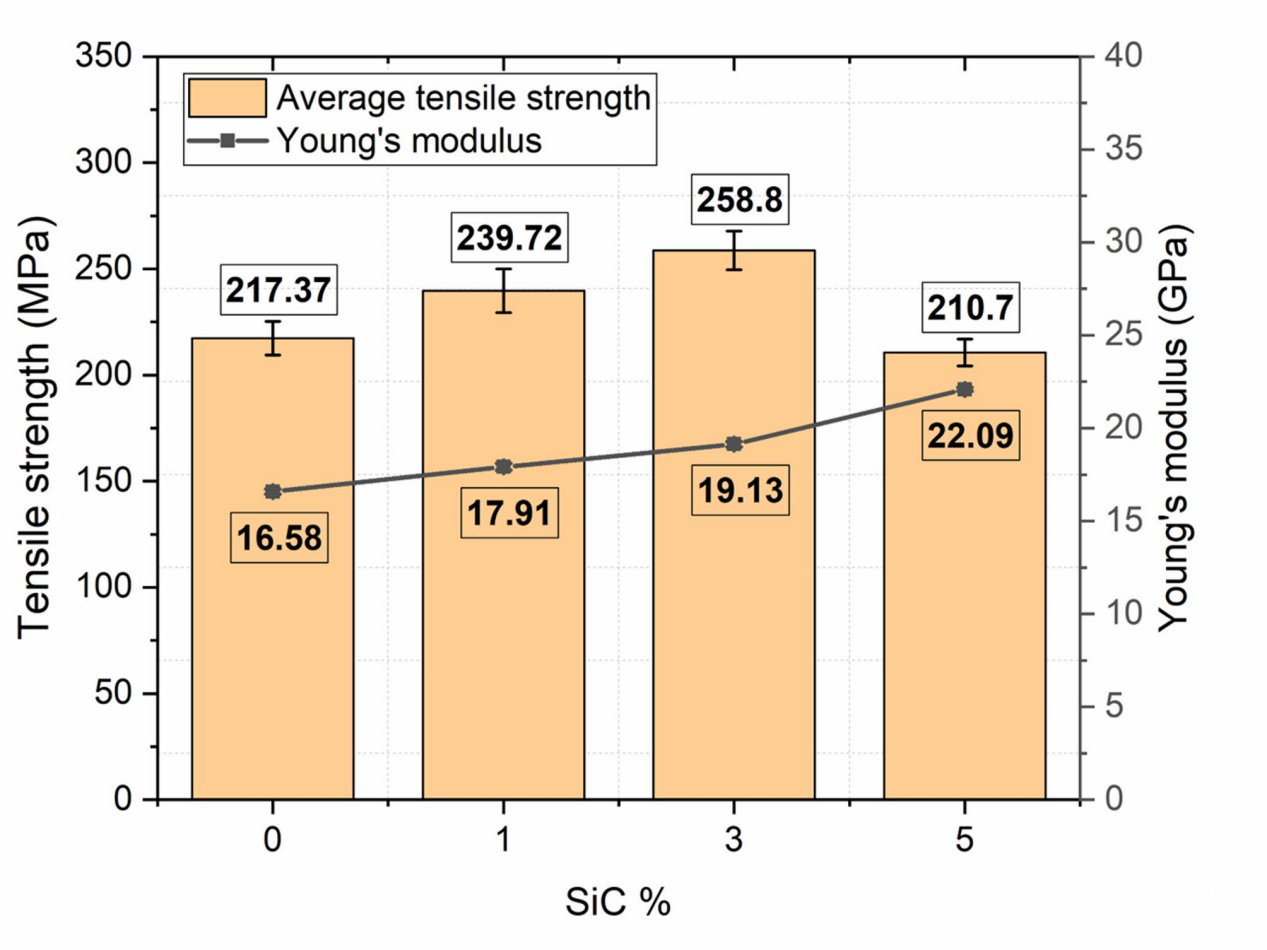
Tensile strength and Young’s modulus of the composites.

In summary, the tensile results reaffirm the critical importance of optimizing filler content. The 3 wt% SiC reinforcement emerged as the most effective composition, providing the best combination of tensile strength, ductility, and stiffness, with an increase of 19.05% and 15.37% in tensile strength and modulus, respectively, compared to 0 wt% SiC composites. Even though stiffness continued to increase with higher SiC content, the loss in strength and strain capacity at 5 wt% indicates the onset of material brittleness, highlighting a performance trade-off beyond the optimal reinforcement level. Figure 11 presents the tensile strength and Young’s modulus of the composites.

**Fig. 12:**
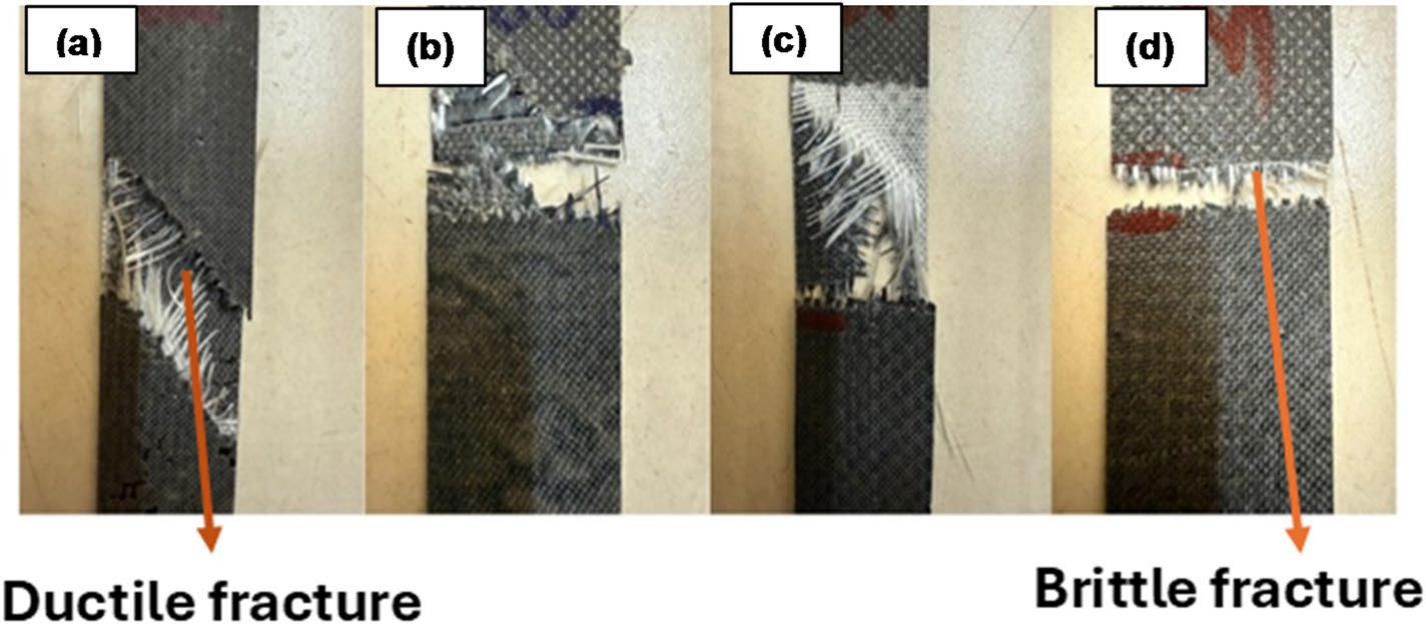
Tensile fractured specimens of (**a**) 0% (**b**) 1% (**c**) 3% and (**d**) 5 wt% of SiC.

The fractured tensile specimens of the glass–carbon hybrid composites with different SiC weight percentages (0%, 1%, 3%, and 5%) are shown in Fig. 12. At 0 wt% SiC, the specimen exhibits ductile fracture, which is supported by extensive fibre pull-out and an inclined fracture surface. The inclined fracture indicates that the specimen experienced significant shear deformation before the fracture, resulting in fibre pull-out. In the absence of SiC nanoparticles, the matrix allows fibers to bridge cracks and absorb energy, resulting in a more gradual and energy-dissipating failure process. At 1 wt% SiC, the fracture still displays ductile features, but the fiber pull-out and fraying are somewhat reduced. This suggests that the addition of a small amount of SiC nanoparticles has started to enhance the fiber–matrix interface, leading to improved load transfer between the matrix and fibers. The composite still retains some toughness, but the increased interfacial bonding begins to shift the failure mode toward a more cohesive and stronger response. At 3 wt% SiC, the specimen demonstrates the most balanced fracture surface. Here, the fiber pull-out is minimized, and the fracture appears more uniform and less jagged compared to the previous specimens. This indicates that the optimal SiC content has significantly improved the matrix reinforcement and fiber–matrix adhesion, resulting in the maximum tensile strength observed among all specimens. The improved bonding enables efficient stress transfer, allowing the composite to withstand higher loads before failure, while still preventing a completely brittle break. At 5 wt% SiC, the specimen shows a brittle fracture, characterized by a clean, flat break with minimal fiber pull-out or matrix deformation. This failure mode is associated with excessive SiC nanoparticle addition, which can lead to nanoparticle agglomeration and the formation of stress concentration sites. These defects weaken the composite and cause a sudden, catastrophic failure with little energy absorption. Thus, while increasing SiC content initially enhances strength, too much SiC leads to embrittlement and reduced mechanical performance.

**Fig. 13:**
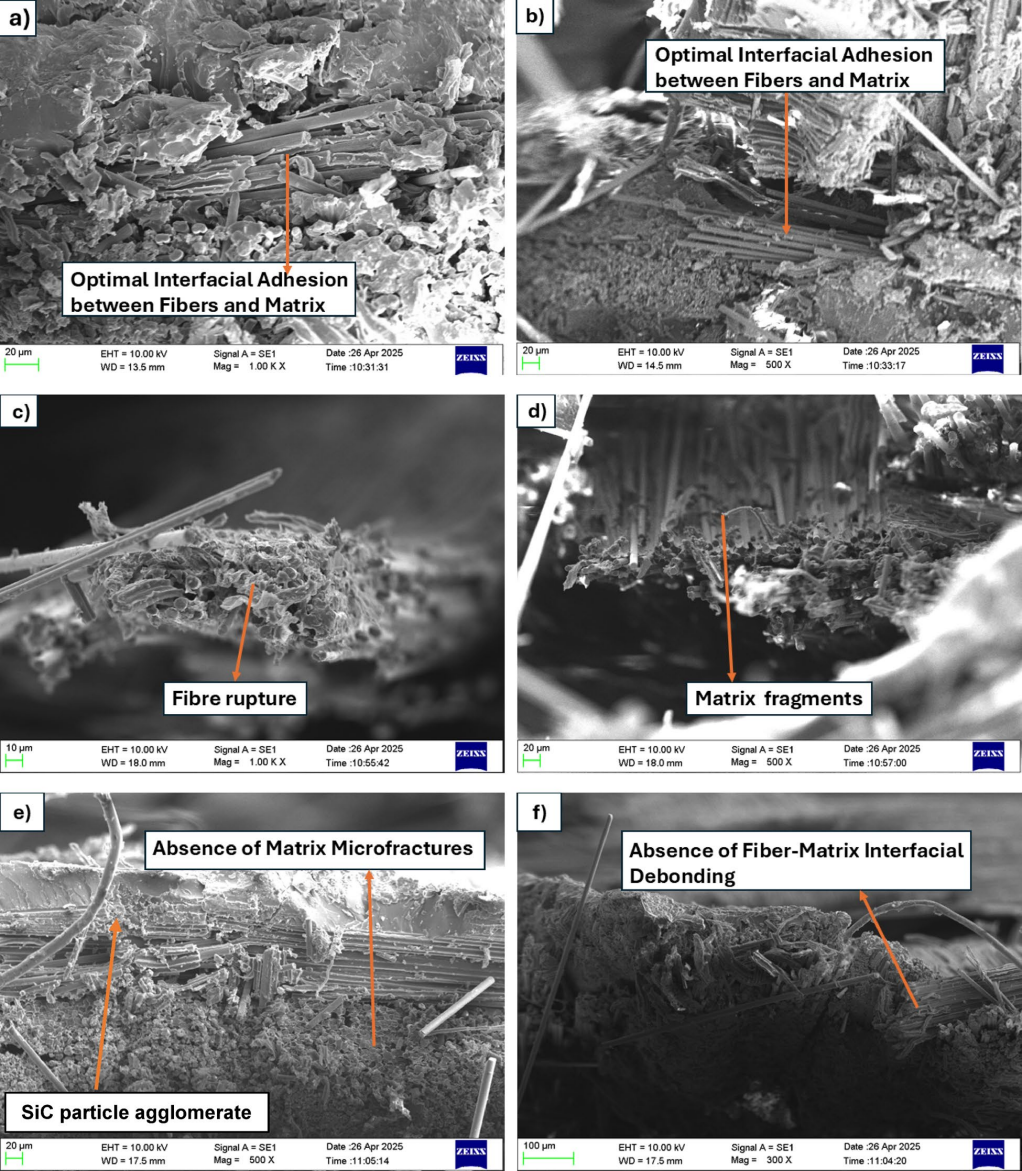

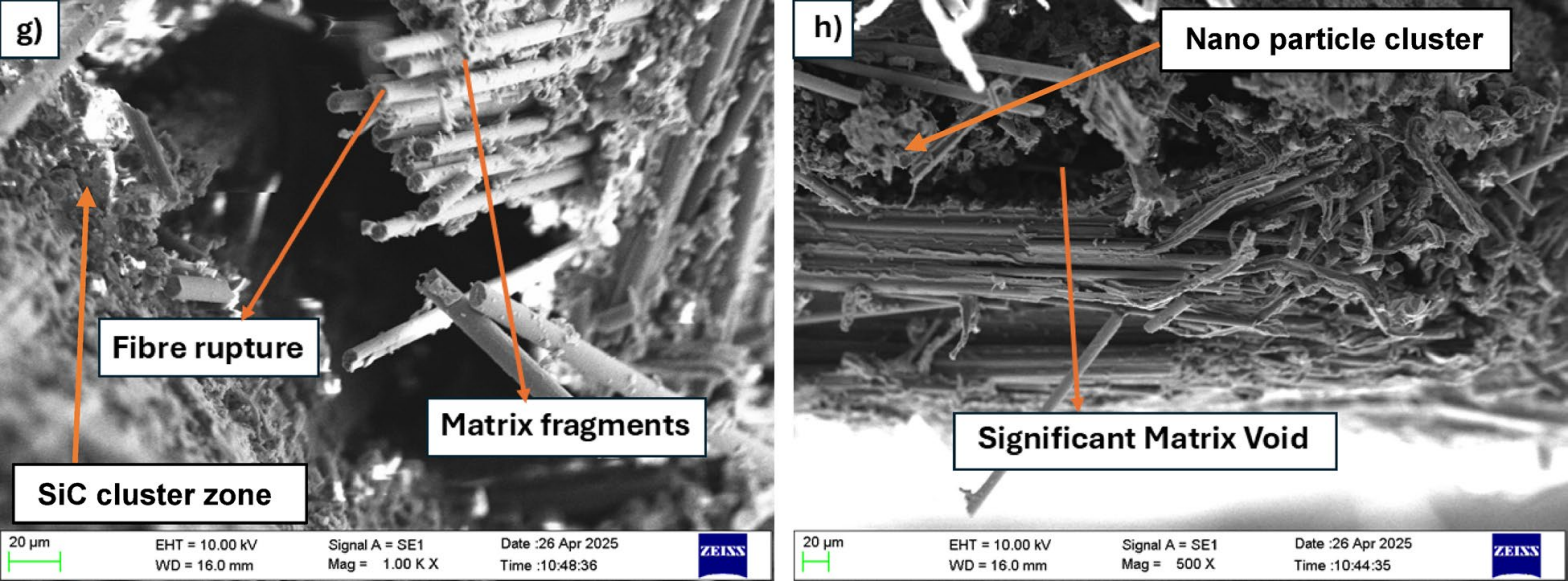
SEM images of tensile fractured specimens. (a, b) 0 wt% (c, d) 1 wt% (e, f) 3 wt% (g, h) 5 wt%.

The SEM micrographs offer insightful information about the microstructural origin of the composite laminates with varying SiC filler concentrations and their influence on fiber–matrix interaction, fracture behavior, and overall mechanical performance. In this study, the dispersion quality and agglomeration behavior of SiC nanoparticles were validated through Scanning Electron Microscopy (SEM) analysis. The micrographs provided direct visual confirmation of uniform nanoparticle distribution at 3 wt% SiC and the onset of cluster formation and agglomeration at higher concentrations (5 wt%). No separate quantitative image analysis technique was employed, as SEM observation was found sufficient to qualitatively assess the dispersion and clustering characteristics. In Fig. 13.(a, b) (0 wt% SiC), the composite surface reveals densely packed fiber bundles with minimal signs of fiber–matrix debonding or matrix cracking. The interface between the fibers and matrix appears uniform and well-bonded, indicating that the baseline composite possesses decent interfacial adhesion. There is no visible fiber pull-out or micro-void formation, which implies that the load transfer between the matrix and fibers is relatively efficient in the absence of SiC fillers. This microstructural stability is reflected in the moderate mechanical strength observed in the corresponding tensile results. Figure 13(c, d) (1 wt% SiC), noticeable changes begin to appear in the composite morphology. The micrograph shows regions of fragmented matrix surrounding partially pulled-out fibers and local fiber breakage. Although some SiC particles are expected to reinforce the matrix and improve load transfer, the dispersion at this stage may not yet be uniform, leading to localized stress accumulation and microcracking. The presence of pulled-out fibers indicates partial debonding at the interface, and although the mechanical strength shows improvement compared to the neat composite, the damage features highlight the limitations of low filler content in fully stabilizing the matrix under stress.

Figure 13(e, f) (3 wt% SiC) exhibits the most optimized and structurally sound morphology among all samples. The fibers are seen to be fully embedded within a uniform and continuous matrix, and the dispersion of SiC particles appears homogeneous, contributing to enhanced interfacial bonding. There are minimal signs of cracking, fiber pull-out, or matrix degradation. The improved microstructural integrity facilitates better load distribution and energy dissipation during deformation, which correlates directly with the highest tensile strength and strain values observed. The SiC fillers at this concentration act effectively as crack arrestors and enhance the matrix toughness without compromising its cohesion with the fibers. In contrast, Fig. 13(g, h) (5 wt% SiC) reveals a significant decline in microstructural quality. The micrograph clearly shows large voids, matrix fragmentation, and multiple fiber fractures. The excessive incorporation of SiC particles likely leads to agglomeration and uneven dispersion, which disrupts the matrix continuity and introduces internal stress concentrators. These defects serve as favorable sites for crack initiation and propagation under loading conditions. Additionally, the voids and fiber breakage decreases the overall load-carrying capacity of the composite, explaining the sharp drop in mechanical performance at higher filler loading. The weakened interfacial bonding and brittle nature of the overfilled matrix contribute to premature failure mechanisms.

In summary, the SEM analysis confirms a strong correlation between filler content and microstructural behavior. The addition of SiC up to an optimal level (3 wt%) enhances fiber–matrix bonding, reduces crack propagation, and improves mechanical properties. However, excessive addition beyond this threshold adversely affects the composite’s structural integrity due to filler agglomeration, matrix discontinuity, and fiber damage. Thus, a balanced filler content is critical for achieving superior mechanical performance in hybrid composites. A comparison with other nanocomposite systems reported in the literature further validates the present findings. A study reported an increase of approximately 40–60% in tensile strength and modulus for graphene-oxide-functionalized epoxy composites, which are of a similar magnitude to the 19–25% improvements observed in this work at 3 wt% SiC^36^. These results indicate that SiC nanoparticles provide reinforcement efficiencies comparable to other nanofillers such as graphene oxide, Al₂O₃, and CNTs when optimally dispersed, thereby supporting the effectiveness of SiC as a viable reinforcing phase in hybrid laminates.

### Flexural behaviour

Table 6 shows the flexural properties of composite samples containing different amounts of SiC, specifically 0%, 1%, 3%, and 5% by weight. The results clearly show that adding SiC has a noticeable effect on how the material behaves, especially in terms of its maximum flexural strength, strain at failure, and Young’s modulus.

**Table 6:** Flexural test results.

SiC wt%	Ultimate strength (MPa)	Failure strain	Young’s modulus (GPa)
0	253.93 ± 7.62	0.0173	13.37 ± 0.62
1	269.72 ± 8.09	0.0156	14.85 ± 0.67
3	292.58 ± 8.78	0.0141	16.79 ± 0.76
5	259.50 ± 7.79	0.0159	13.81 ± 0.71

**Fig. 14:**
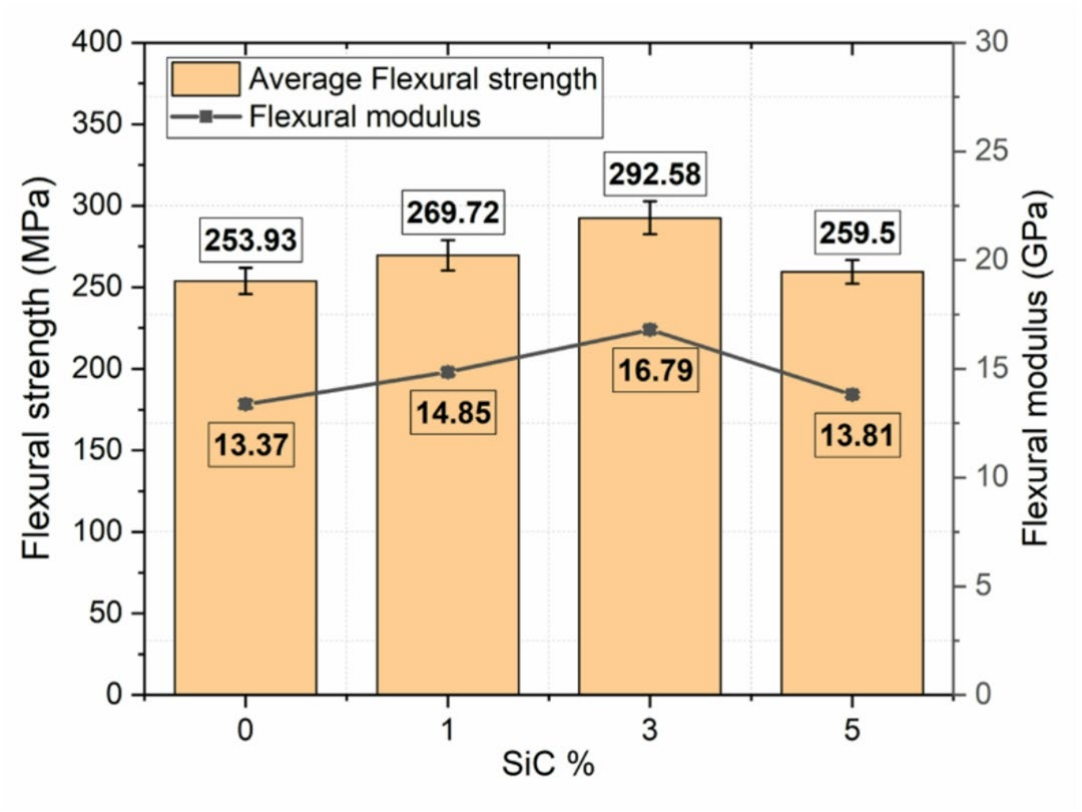
Flexual stress and modulus at different SiC wt%.

**Fig. 15:**
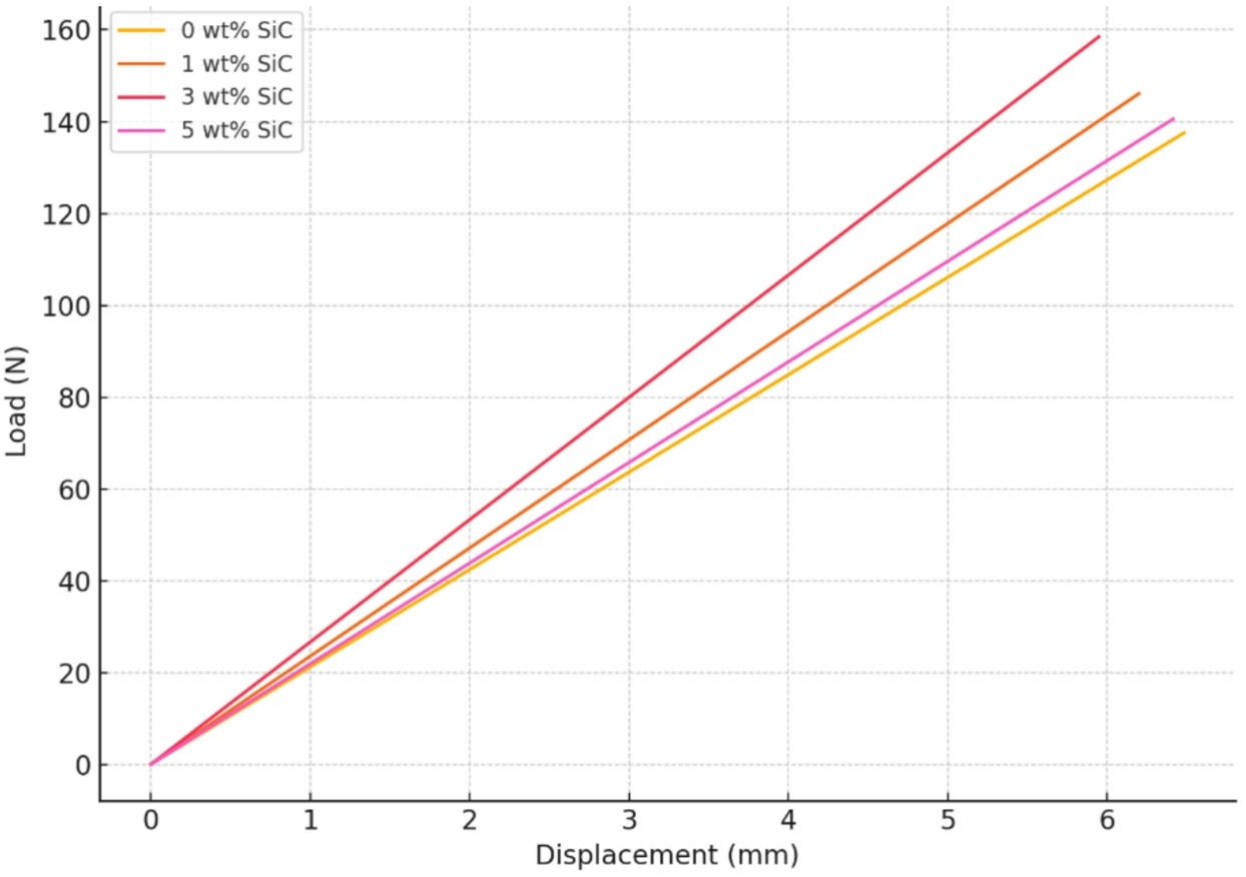
Flexural load vs. displacement graph.

The composite with 0% SiC showed an ultimate flexural strength of 253.93 MPa, a failure strain of 0.0173, and a Young’s modulus of 13.37 GPa, which was used as the reference point for comparison. When 1% SiC was added, there was a clear improvement in mechanical performance. The ultimate strength increased to 269.72 MPa, and the Young’s modulus increased to 14.85 GPa, indicating a stiffer material. However, the failure strain dropped slightly to 0.0156, which is expected as the added SiC particles make the composite more rigid and less flexible. A remarkable improvement was observed at 3 wt% SiC, where the composite exhibited the highest ultimate strength of 292.58 MPa and the maximum Young’s modulus of 16.79 GPa. There was an enhancement of 15.22% in flexural strength and 25.57% in flexural modulus values with reference to 0 wt% SiC composites. This indicates that the addition of 3% SiC provides an optimal balance of strength and stiffness, likely due to efficient stress transfer between the matrix and the well-dispersed SiC particles. The failure strain at this composition, however, was slightly reduced to 0.0141, consistent with the trend that increasing stiffness can lead to a reduction in ductility. Interestingly, when the SiC content was further increased to 5 wt%, a decline in both strength and stiffness was observed. The ultimate strength dropped to 259.5 MPa, and Young’s modulus decreased to 13.81 GPa. Although still higher than the unreinforced baseline, this decrease from the peak values at 3 wt% suggests the onset of negative effects such as particle agglomeration or poor interfacial bonding, which can act as stress concentrators and reduce overall load-bearing capacity. The failure strain for this composition was 0.0159, remaining within a similar range but not indicating any major improvement in ductility. Figure 14 shows the flexural strength and modulus of SiC hybrid composites. The representative load versus displacement curves for different SiC weight percentages exhibit a linear elastic trend up to the failure point as shown in Fig. 15, confirming elastic bending behaviour within the test range. Among all compositions, the 3 wt% SiC laminate shows the steepest slope and highest ultimate load, indicating superior stiffness and load-carrying capacity. The increased slope corresponds to the enhanced flexural modulus due to improved interfacial bonding and effective stress transfer from matrix to reinforcement. In contrast, the 5 wt% SiC sample displays a lower slope and reduced ultimate load, suggesting that excessive filler content leads to agglomeration and microstructural defects that compromise bending performance.

**Fig. 16:**
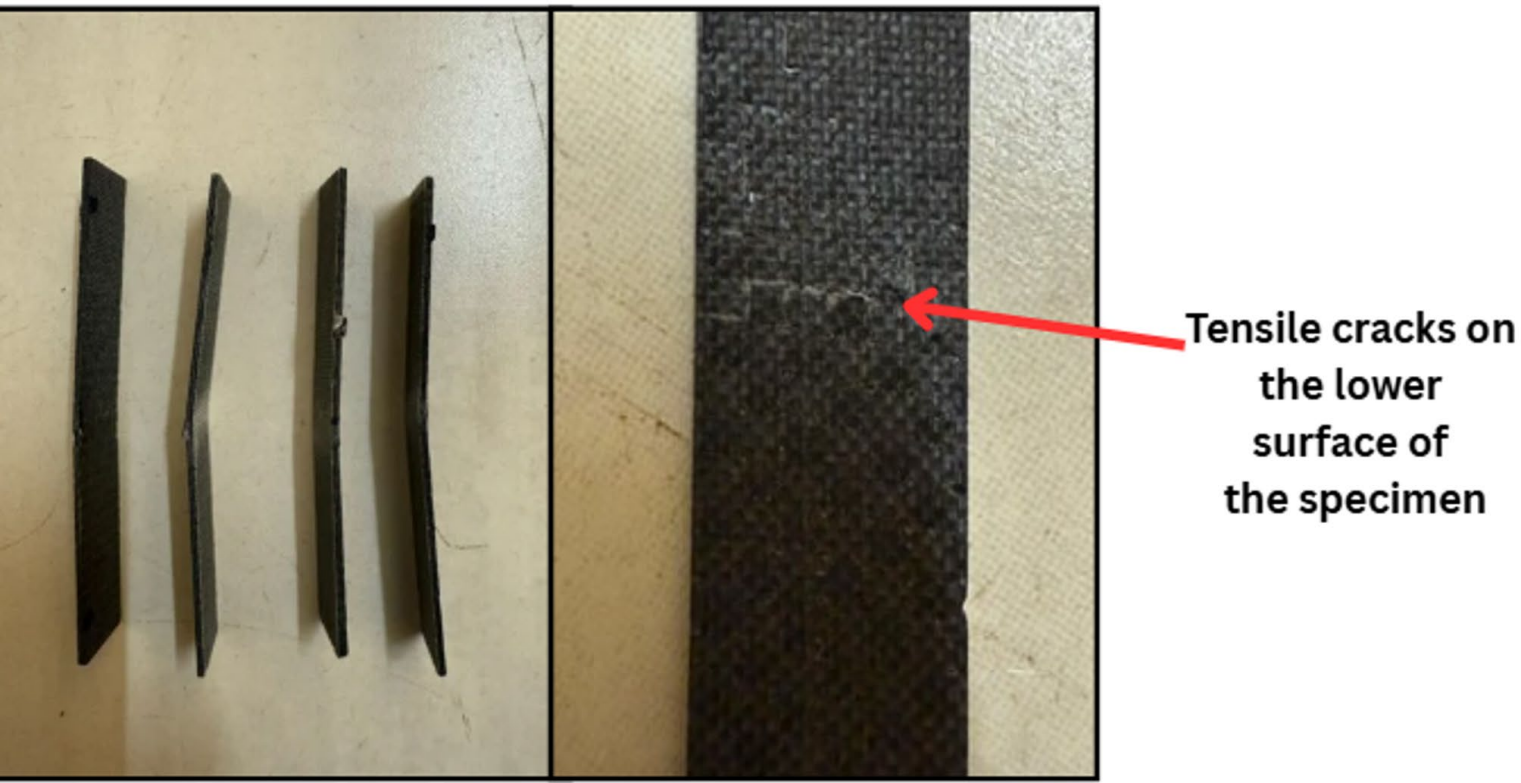
Fractured flexural specimens (**a**) Side view (**b**) cracks on the tensile surface.

In summary, the results clearly show that the addition of SiC reinforcement enhances the flexural properties of the composite, with 3 wt% SiC demonstrating the most favorable mechanical performance. Beyond this concentration, further addition of SiC appears to result in diminishing returns due to potential defects in the microstructure. These findings underscore the importance of optimizing filler content to achieve superior mechanical properties in composite systems. The fractured specimens from the bending test, as shown in Fig. 16, exhibit typical flexural failure characteristics. All samples display visible bending and cracking, indicating the onset of failure under three-point loading. Notably, Fig. 16b highlights tensile cracks on the lower surface of the specimen, which is consistent with the expected tension-side failure in flexural loading. These cracks confirm that the material failed due to tensile stress on the bottom face, where the bending moment induces maximum tension. The absence of severe delamination suggests good interlaminar bonding, with failure primarily governed by matrix cracking and fiber breakage.

### Impact behaviour

Table 7 presents the impact test results obtained for composite specimens reinforced with varying weight percentages of silicon carbide (SiC) particles, ranging from 0% to 5%. The results indicate a clear influence of SiC content on both the absorbed impact energy and the corresponding impact strength of the composite laminates. At 0 wt% SiC, the absorbed energy was recorded as 1.26 J, and the impact strength was 63.07 kJ/m^2^. With the introduction of SiC reinforcement at 1 wt%, the absorbed energy increased to 1.28 J, and a marginal improvement in impact strength of 63.96 KJ/m^2^ was observed. This suggests that even a small addition of SiC enhances the material’s ability to absorb and dissipate impact energy, likely due to improved stiffness and microstructural reinforcement effects imparted by the SiC particles.

**Table 7:** Impact test results.

SiC wt%	Absorbed energy (Joules)	Impact strength (KJ/$\:{m}^{2}$)
0	1.26	63.07 ± 1.89
1	1.28	63.96 ± 1.92
3	1.36	67.90 ± 2.04
5	1.15	57.29 ± 1.72

**Fig. 17:**
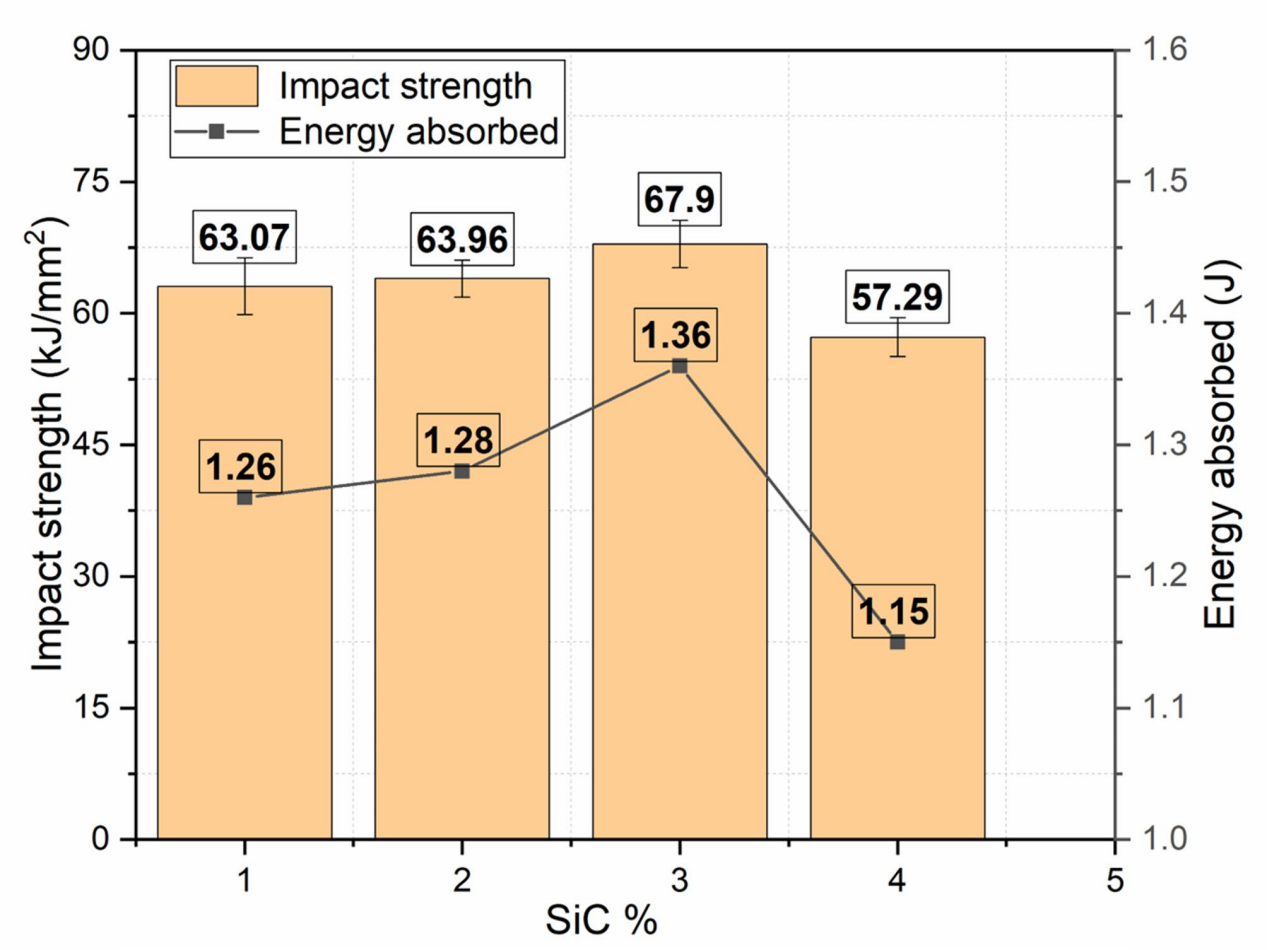
Impact strength and absorbed energy of SiC hybrid composites.

The most significant enhancement in impact performance was observed at 3 wt% SiC, where the composite recorded the highest absorbed energy of 1.36 J and an impact strength of 67.90 kJ/m^2^. This substantial improvement can be attributed to the optimal dispersion of SiC particles within the matrix, which enhances the fiber–matrix interfacial bonding and leads to better energy dissipation during sudden impact events. The SiC particles act as stress arresters, hindering crack propagation and allowing the composite to sustain higher energy before failure. This behavior reflects an ideal balance between stiffness, toughness, and particle distribution within the composite structure. However, as the SiC content was further increased to 5 wt%, a noticeable drop in impact performance was observed. The absorbed energy decreased to 1.15 J, and the corresponding impact strength dropped significantly to 57.29 kJ/m^2^. This decline can be attributed to the agglomeration of SiC particles at higher concentrations, which often leads to the formation of stress concentration sites and microvoids within the matrix. Such microstructural inconsistencies hinder effective load transfer and promote brittle failure mechanisms under dynamic loading conditions. Additionally, the excessive presence of rigid SiC particles may restrict the deformation capabilities of the matrix, thereby reducing the overall toughness of the composite. While the variatoin in the impact strenght and absorbed energy across the weight percentages is illustrated in Fig. 17.

**Fig. 18:**
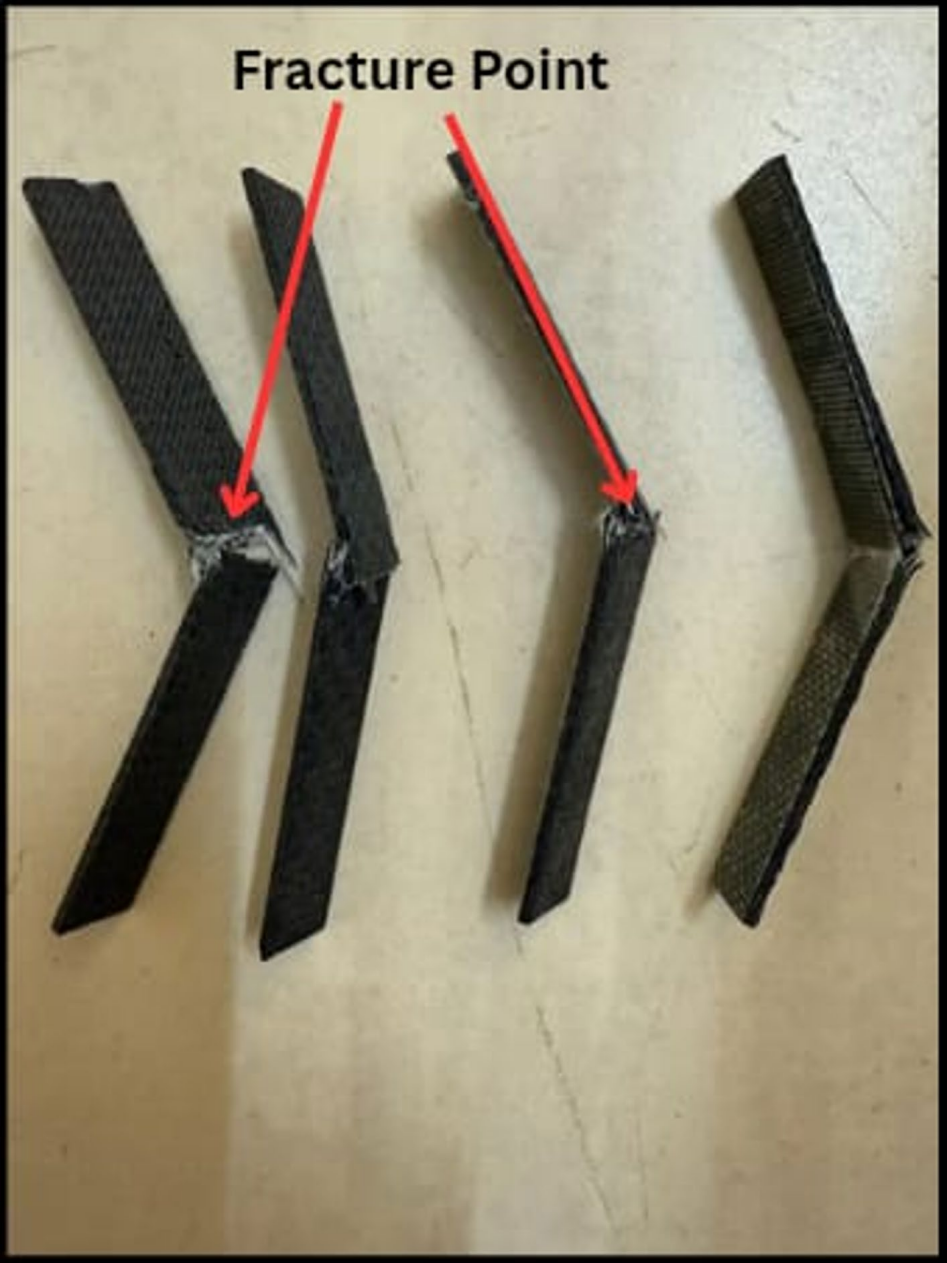
Fractured impact test specimens.

In summary, the impact results clearly demonstrate that the incorporation of SiC particles significantly influences the impact resistance of the composite system, with 3 wt% SiC showing the most favorable outcome. This composition provides the best combination of absorbed energy and impact strength, indicating its potential suitability for applications requiring superior impact performance. These findings also underline the significance of optimizing filler content to achieve desirable mechanical characteristics without compromising the integrity of the laminate structure. Figure 18 depicts the fractured impact specimens.

### Vibrational analysis

The free vibration analysis of the hybrid composites reveals significant variations in dynamic properties with the incorporation of different weight percentages of SiC, as shown in Table 8. The natural frequency and stiffness both show a marked increase with the addition of filler up to 3 wt%, indicating enhanced structural rigidity. Specifically, the 3 wt% SiC composite achieves the highest natural frequency of 22 Hz and a maximum stiffness of 5063.5 N/m, suggesting superior vibrational resistance and load-bearing capability compared to the other formulations. This enhancement can be attributed to the optimal dispersion and bonding of SiC particles within the matrix at this concentration, resulting in improved mechanical interlocking and rigidity.

**Table 8:** Free vibration test results.

SiC wt%	Natural frequency (Hz)	Stiffness (*N*/m)	Logarithmic decay (δ)	Damping ratio (ξ)
0	10.57	1376.14 ± 41.28	0.950	0.150
1	19.0	3776.70 ± 113.3	0.317	0.0505
3	22.0	5063.50 ± 151.9	0.164	0.0262
5	11.24	1556.13 ± 46.68	0.134	0.0214

In contrast, the 0 wt% and 5 wt% composites exhibit lower natural frequencies and stiffness values, indicating inferior dynamic performance. The 5 wt% sample, in particular, shows a significant drop in both parameters, which may result from agglomeration of filler particles or weakened matrix-filler interactions at higher concentrations. Regarding damping characteristics, the 0 wt% SiC composite exhibits the highest damping ratio of 0.15 and a logarithmic decay of 0.95, implying superior energy dissipation capability. However, as the SiC content increases, a noticeable reduction in both damping ratio and logarithmic decay is observed. The 3 wt% and 5 wt% composites show lower damping values, indicating that while stiffness and natural frequency improve, the ability to dissipate vibrational energy decreases. This is a common trade-off in composite materials where stiffness enhancement often comes at the cost of damping capacity.

**Fig. 19:**
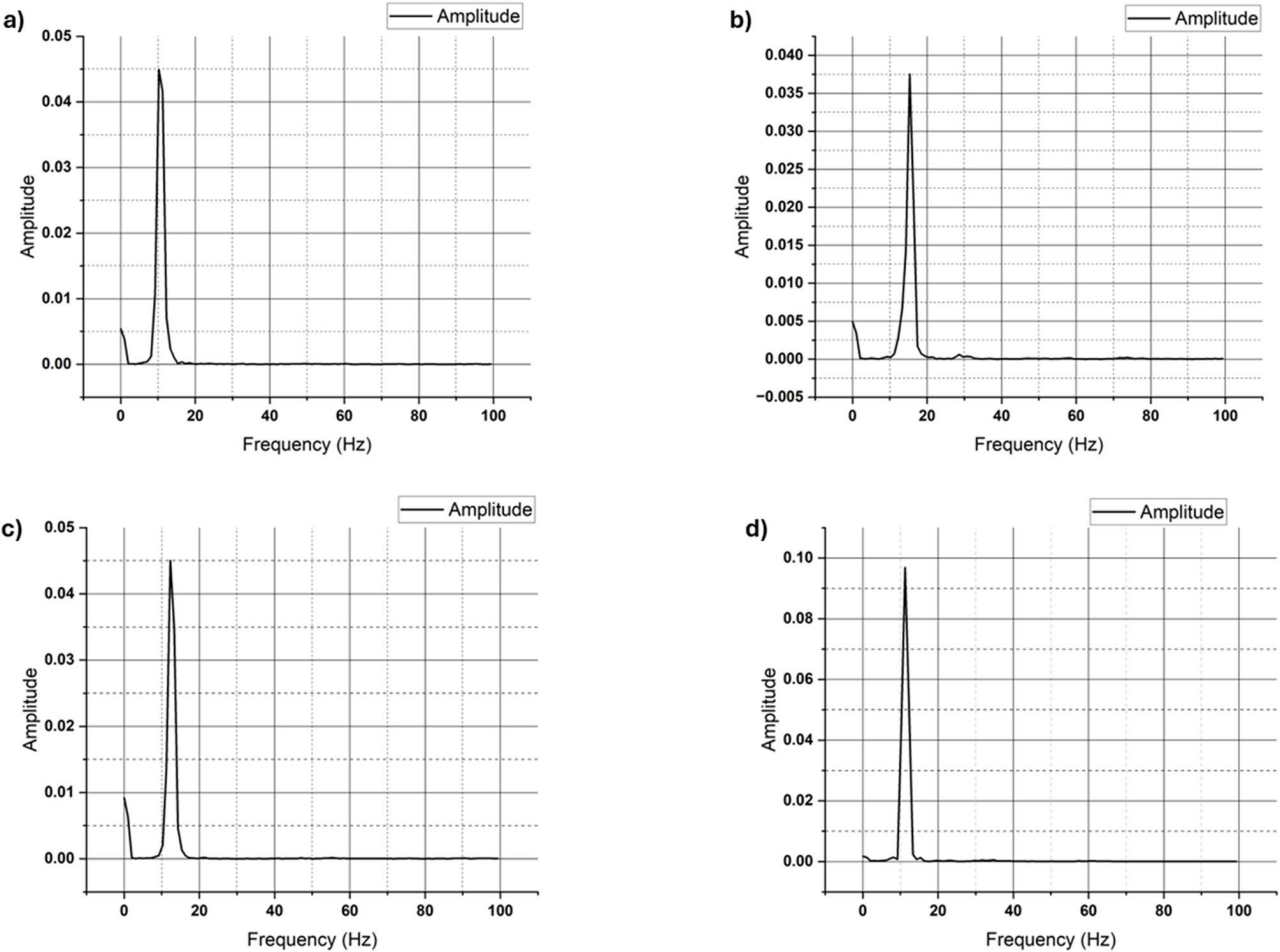
Acceleration amplitude vs. frequency plots of (**a**) 0%, (**b**) 1%, (**c**) 3% and (**d**) 5% SiC hybrid composites.

Overall, the 3 wt% SiC composite offers the best balance between increased stiffness and natural frequency while maintaining acceptable damping characteristics, making it a favorable choice for applications where structural integrity and vibration control are critical. The Fast Fourier Transform (FFT) plots, which depict acceleration amplitude versus frequency, are shown in Fig. 19.

### Sound impedance test

**Fig. 20:**
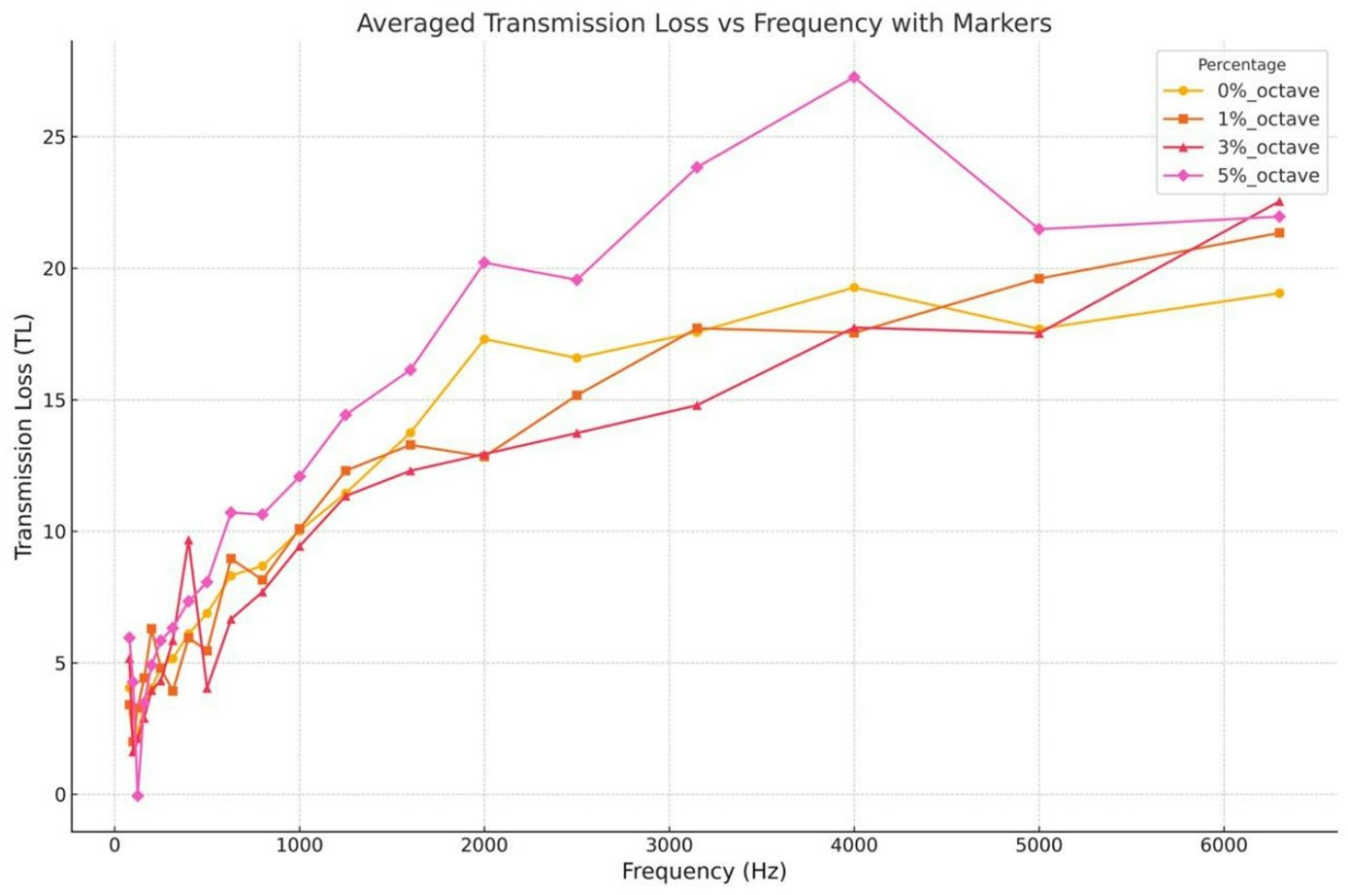
Transmission loss vs. frequency.

The sound absorption characteristics of the hybrid composites, analyzed through Transmission Loss (TL) measurements across a wide frequency range, are illustrated in the Fig. 20. A consistent trend is observed across all compositions—TL values increase with frequency, confirming that the materials exhibit improved sound insulation performance at higher frequencies. Among all the weight percentages tested, the composite containing 5 wt% of SiC demonstrates the most effective acoustic performance, attaining a peak TL of around 27 dB near 4000 Hz. This enhancement can be attributed to the increased internal friction and scattering effects caused by the higher content of SiC particles, which act as barriers to sound wave propagation. The 3 wt% SiC sample also exhibits a notable improvement over the 0% and 1% compositions, especially in the mid-frequency range (1000–4000 Hz), suggesting a balance between effective sound attenuation and material density. Although the 1 wt% sample offers a modest increase in TL compared to the neat matrix (0 wt%), its performance does not match the higher filler-loaded composites. Interestingly, the 5 wt% sample, while superior overall, shows slight fluctuations in TL at higher frequencies, possibly due to filler agglomeration or resonance effects at specific frequencies.The enhanced transmission loss observed with increasing SiC content can be explained by increased internal scattering and impedance mismatch introduced by rigid nanoparticles within the matrix. Higher filler loading increases wave reflection, frictional losses, and tortuosity of sound propagation paths. However, this acoustic benefit occurs at the expense of structural integrity beyond the optimal filler content, highlighting a trade-off between mechanical performance and acoustic damping.

Overall, these results confirm that the incorporation of SiC significantly enhances the acoustic damping capability of the composites, with the 5 wt% formulation yielding the highest sound attenuation across most of the tested frequency range. This makes it a promising candidate for applications requiring effective noise control.

The observed increase in transmission loss with higher SiC content in this study aligns with trends reported in recent natural-fiber acoustic composites. A study showed that acoustic performance in impedance tube testing is strongly influenced by filler-induced scattering and internal friction mechanisms^37^, similar to the behaviour noted in the present carbon–glass/SiC hybrid laminates. The reported work^37^ demonstrated higher sound absorption when the reinforcement increased the tortuosity and energy dissipation pathways, which closely matches the improvement observed in current study 3 wt% and 5 wt% SiC samples. This comparison reinforces that SiC nanoparticles enhance acoustic attenuation through mechanisms comparable to other micro- and nano-reinforced systems.

## 5 conclusion

This study investigated the influence of silicon carbide (SiC) nanoparticles on the mechanical, vibrational, and acoustic performance of carbon–glass/epoxy hybrid composites. The results demonstrate that SiC addition significantly modifies the multifunctional behavior of the laminates, with performance strongly dependent on filler concentration. The summary of the test results is as follows,

- The study demonstrated that incorporating SiC nanoparticles significantly enhanced the mechanical, vibrational, and acoustic characteristics of the hybrid carbon–glass/epoxy composites. The optimal performance was observed at 3 wt% SiC, where the tensile strength increased from 217.37 MPa to 258.80 MPa (19.05% improvement) and the flexural strength improved from 253.93 MPa to 292.58 MPa (15.22% improvement). The tensile modulus increased from 16.58 GPa to 19.13 GPa (15.37% gain), while the flexural modulus increased from 13.37 GPa to 16.79 GPa (25.57% gain). Impact performance also improved, with the impact strength rising from 63.07 kJ/m^2^ to 67.90 kJ/m^2^ (7.65% improvement).
- The improvement in tensile and flexural properties up to 3 wt% SiC can be attributed to enhanced stress transfer facilitated by uniformly dispersed nanoparticles, which act as nanoscale bridges between the epoxy matrix and fiber surfaces. These particles restrict polymer chain mobility locally, increase matrix stiffness, and suppress microcrack initiation at the fiber–matrix interface. At higher filler loading (5 wt%), nanoparticle agglomeration disrupts matrix continuity and creates localized stress concentration zones, which promote premature crack initiation and brittle fracture, outweighing the benefits of increased stiffness.
- Vibrational analysis showed an increase in natural frequency from 10.57 Hz to 22.00 Hz and a reduction in damping ratio from 0.150 to 0.0262, indicating enhanced stiffness and reduced energy dissipation due to better nanoparticle dispersion. Overall, the results confirm that 3 wt% SiC provides the most effective reinforcement, balancing stiffness, strength, and dynamic performance before agglomeration effects dominate at higher filler contents.
- The increase in natural frequency and stiffness with SiC addition up to 3 wt% is primarily governed by the increase in effective elastic modulus of the composite system. The reduction in damping ratio with increasing filler content is associated with constrained polymer chain mobility and reduced viscoelastic energy dissipation within the epoxy matrix. While the neat composite exhibits higher damping due to matrix-dominated energy dissipation, the presence of rigid SiC nanoparticles shifts the response toward stiffness-controlled behavior, resulting in reduced damping capacity at higher filler concentrations.
- Scanning Electron Microscopy (SEM) analysis further supported these findings, showing a well-integrated microstructure with minimal defects and effective filler distribution at the optimal concentration.
- Increasing the SiC content to 5 wt% resulted in a decline in mechanical performance despite marginal gains in stiffness and acoustic attenuation. This reduction is primarily associated with nanoparticle agglomeration, matrix discontinuity, and the formation of stress concentration sites, which promoted brittle failure mechanisms.
- Overall, the 3 wt% SiC formulation emerged as the most balanced in terms of mechanical strength, energy absorption, stiffness, and acoustic damping, making it a promising candidate for structural and functional applications in aerospace, automotive, and acoustic insulation fields.

## Data Availability

The datasets used and/or analysed during the current study available from the corresponding author on reasonable request.
